# Unveiling sequence-agnostic mixed-chemical modification patterns for splice-switching oligonucleotides using the NATURA platform

**DOI:** 10.1016/j.omtn.2024.102422

**Published:** 2025-01-07

**Authors:** Tommaso Tabaglio, Taniya Agarwal, Wei Yuan Cher, Jin Rong Ow, Ah Keng Chew, Priscila Yun Qian Sun, Raja Sekhar Reddy Gurrampati, Hongfang Lu, Praveena Naidu, Hong Kai Ng, Xavier Le Guezennec, Shi Yan Ng, Manikandan Lakshmanan, Ernesto Guccione, Keng Boon Wee

**Affiliations:** 1 Institute of Molecular and Cell Biology (IMCB), Agency for Science, Technology and Research (A∗STAR), Singapore, Singapore; 2 Center for OncoGenomics and Innovative Therapeutics (COGIT), Center for Therapeutics Discovery, Department of Oncological Sciences and Pharmacological Sciences, Tisch Cancer Institute, Icahn School of Medicine at Mount Sinai, New York, NY, USA

**Keywords:** MT: Oligonucleotides: Therapies and Applications, antisense oligonucleotides, nucleic acid therapeutics, reporter system, nucleic acid chemical modifications, mixmers, splice-switching oligonucleotides, splice modulation

## Abstract

Chemical optimization of ribose has significantly advanced nucleic acid therapeutics (NATs) by improving the stability, specificity, and safety of therapies like small interfering RNAs, CRISPR-Cas9 guide RNAs, and GAPmers. Recent research has extended this approach to splice-switching oligonucleotides (SSOs), which target splicing events. Our study identifies a set of mixed-modification patterns—combining 2′-O-Methyl, 2′-MethOxyEthyl, 2′-Locked Nucleic Acid, and 2′-Constrained Ethyl ribose moieties (2′OMe, 2′MOE, LNA, and cET)—that enhance SSO potency. We term this strategy lateral mixed positional configuration, which improves SSO efficacy across various sequences and could reduce the trial-and-error process in SSO development. This advancement is supported by NAT Unlabeled Reporter Assay (NATURA), a novel platform for high-throughput quantification of NATs' functional delivery and potency. NATURA uses a reporter gene system and a comprehensive sequence library to test modifications and delivery methods, validated in a transgenic mouse model. This approach aims to accelerate NAT development and address challenges in delivering these therapies to patients.

## Graphical abstract

**Figure:**
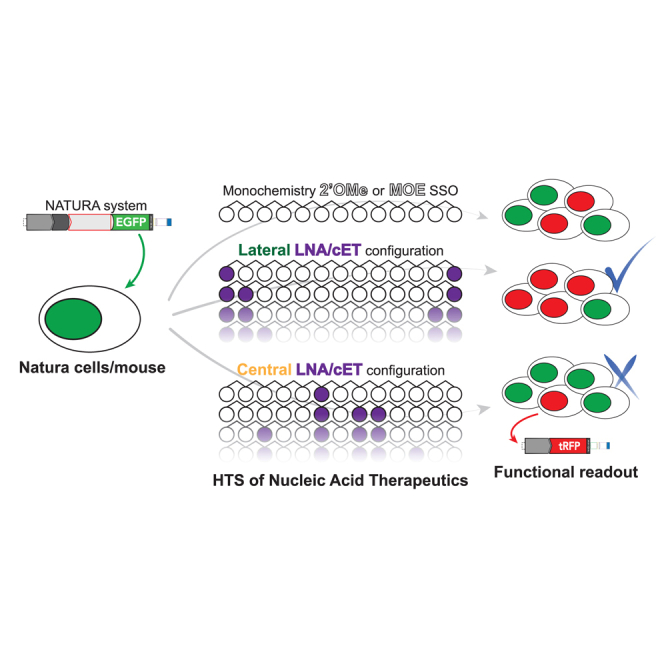

## Teaser

NATURA, a breakthrough platform for NAT functional assays, reveals that the lateral MPC of ribose modifications consistently delivers the most effective SSOs. This finding simplifies and accelerates the development of NATs by optimizing oligo performance.

## Introduction

The broad plethora of compounds embraced under the label of nucleic acid therapeutics (NATs) have helped to overcome multiple medical challenges in the recent decade, especially improving the lives of patients affected by debilitating genetic diseases.^1^^,^^2^^,^^3^^,^^4^^,^^5^ This group of therapeutic modalities encompasses formulations that contain a modified or natural nucleic-acid payload, such as small interfering RNA (siRNA), CRISPR systems (with their guide RNAs [gRNAs]),^6^ and antisense oligonucleotides (ASOs), which among others include GAPmers and steric blocking splice-switching oligonucleotides (SSOs).^7^ Despite the initial success in the clinic, the poor bioavailability and/or potency of NATs besets their relevance for most clinical indications. Pioneered decades ago, the incorporation of numerous chemical modifications on both the sugar moiety and inter-nucleotide linkage of an ASO have not only bestowed resistance to nucleases but has also significantly enhanced its therapeutic efficacy. On the other hand, ligand conjugations (e.g., GalNAc) and encapsulation-based formulations (e.g., lipid nanoparticles) have solved NAT delivery to hepatocytes and liver respectively. Given the plethora of chemical modifications and delivery systems, the need for reliable methods to quantify the functional activity of NATs motivated the development of various reporter systems.

Current state-of-the-art methods to test for NATs’ uptake and efficacy can be grouped as labeled- or unlabeled-based approaches. For the former, a fluorophore is chemically conjugated to a NAT and used as an indicator of cell uptake which can be evaluated in high-throughput with fluorescence-based assays such as flow cytometry and confocal microscopy. This approach presents two noteworthy challenges. First, fluorophore conjugation may interfere not only with the delivery agents but also with the processing and target engagement activities of the NAT. It also reduces available sites within the NAT for conjugation with cell-targeting ligands. Second, detection of intracellular fluorescence does not necessarily indicate the continued integrity and activity of the NAT (namely, its functional activity or delivery). *In situ* hybridization^8^ or chemical modification-specific antibodies^9^ have been used to detect delivery of unlabeled oligonucleotides *in vivo*, but these techniques are yet not quantifying the NAT’s functional delivery or efficiency either. These limitations are circumvented in some other unlabeled-based approaches by using the abundance of either an endogenous (e.g., MALAT1)^10^ or a reporter (i.e., EGFP or luciferase)^11^ gene to quantitate functional activity of a NAT that modulates the gene target. However, as the inherent variability in the reporter gene expression is not accounted for, reliable quantitation and comparison of functional delivery and potency among cells and across tissues can be challenging. Furthermore, the approach is prone to false positives when the NAT or its delivery agent has some inherent toxicity or interferes with the transcription or translation machinery (e.g., upon TLR activation).^12^ While the expression of one or more housekeeper genes can be used as a normalization control, it is both tedious and challenging to reliably obtain data for every cell, notwithstanding the likelihood that the relative expression of the reporter and housekeeper genes is not invariant among individual cells and across tissues. Dual reporter systems^13^ have been deployed to account for this variability, but the use of two different promoters can hinder the reliability of the measurements due to the differential expression of the reporters upon different biological conditions.

This study describes a new reporter system that, in addition to incorporating the advantages of both aforementioned approaches while overcoming their limitations, enables the investigation of NATs’ sequence (in)dependency of their chemical modifications and delivery systems. In addition, we wanted a single system being able to measure functional delivery of all the major NATs (siRNAs, gRNAs, and ASOs). The NAT Unlabeled Reporter Assay (NATURA) platform encompasses a reporter and an accompanying NAT tool library modulating the former. Its transcriptional and splicing unit (hereafter referred to as the NATURA gene) is an artificial reporter gene, whose transcripts are used as molecular readouts to quantitate functional cellular uptake and potency of NATs. The inherent variability in gene expression is addressed by designing the reporter to express three spliced isoforms, each translating to a distinct protein, and using the relative abundance of the isoforms as the quantitative readout for functional delivery and potency of a NAT. Through the selection of reporter proteins detectable by fluorescence and luminescence methods, the system is high-throughput and is adaptable for interrogation at cellular and tissue levels, and in live animals. The associated NAT tool library consists of multiple sequence counterparts of siRNAs, GAPmer, SSOs, and CRISPR-gRNA; each efficiently modulates the relative isoforms expression, which enables the investigation of sequence dependency. These new features, other than addressing current limitations, can potentially expedite the development and validation of technologies for advancing NAT bioavailability.

The concomitant use of locked nucleic acid (LNA) and 2′-O-methyl (2′OMe), or combination of 2′OMe and LNA moieties (OML) for short, in an SSO was observed to affect its potency.^14^^,^^15^^,^^16^^,^^17^^,^^18^^,^^19^ For instance, after screening more than a hundred OML mixmers over five overlapping sequences, Van Deutekom et al. observed that, for a single SSO sequence, mixmers having sugars substituted with the two moieties at any of the four mixed positional configurations (MPCs) they tested were more potent than the uniformly 2′OMe-modified version. We deployed the NATURA system to quantitate the absolute potencies of SSO mixmers whose sugars were substituted with LNA and 2′OMe or 2' MethOxyEthyl (2'MOE) in high throughput. Specific rules of MPCs that resulted in striking potencies and reduced cell toxicity, as compared with the uniformly modified counterparts, were discovered. Further investigation showed that they were not only agnostic to SSO sequences but are extensible to the (*S*)-cET moiety. Besides eliminating the need to screen many MPC permutations,^3^ the existence of such rules warrants further studies on other dual or higher-order sugar moiety combinations that have not been heretofore explored.

## Results

### NATURA reporter gene

**Figure 1:**
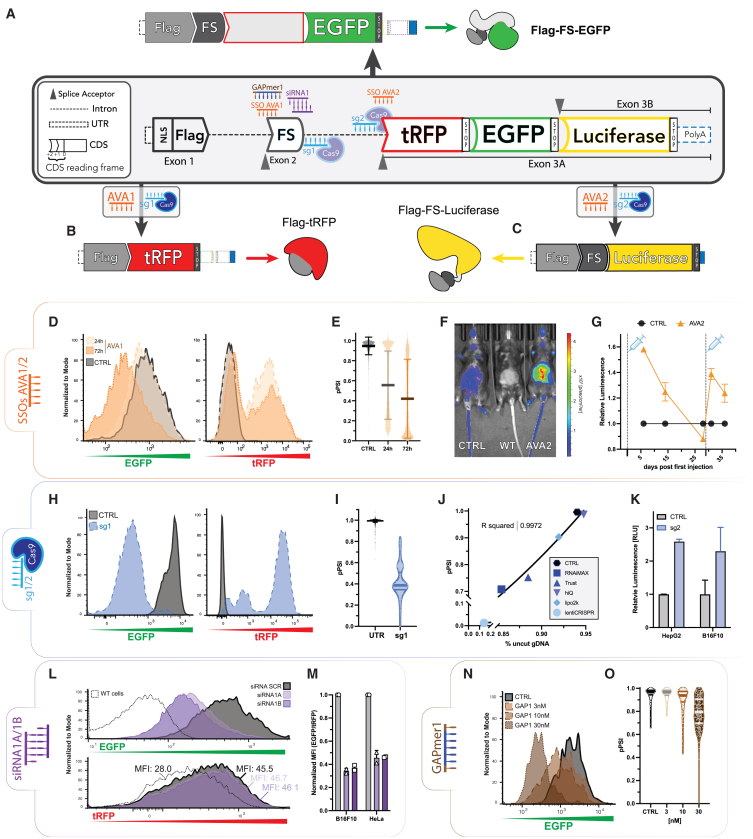
The NATURA gene and its RNA-therapeutics toolbox (A) NATURA reporter gene schematics depicted in the gray rectangle. The “EGFP isoform” is the major transcript expressed. (B) The tRFP isoform. Exons 1 and 3A are spliced in and the resulting transcript is in frame for tRFP, induced upon splice-modulation of FS by AVA1 SSO or CRISPR guide sg1. (C) The Luciferase isoform. Exons 1, FS and 3B are spliced in and the resulting transcript is in frame for Luciferase. It is produced upon skipping exon 3A with AVA2 SSO or sg2 gRNA. (D) Flow cytometry profiles of NATURA-transfected CT26 cells. EGFP (left) and tRFP (right) fluorescence was measured after 24 and 72 h for untreated (CTRL) and 25 nM of AVA1 transfected cells. (E) Single-cell pPSI of FS exon calculated from the data shown in (D). (F) *In vivo* luciferase assay of wild type (WT) and NATURA expressing mice, injected subcutaneously with PBS (CTRL) and 25 mg/kg of AVA2 (AVA2) at 48 h. (G) Untreated-normalized luminescence of NATURA expressing mice injected subcutaneously with 25 mg/kg of AVA2 on day 0 and day 28 over a period of 35 days. (H) Flow cytometry of NATURA-transfected CT26 cells (CTRL) transduced with lentiCRISPRv2-sg1 and selected with puromycin for 5 days. (I) pPSI calculated on single cells from the experiment in (H). (J) Correlation between percentage of uncut genomic DNA (gDNA) and average FS pPSI of NATURA-positive CT26 cells transfected with Cas9/tracrRNA/sg1 using different commercial transfection reagents. Non-linear regression analysis (carriable slope, four parameters, least-squares regression). (K) Relative luminescence of two NATURA positive cell lines, HepG2 and B16F10, transfected with ALT+R Cas9/tracrRNA/sg2 (RNAiMAX). (L) Flow cytometry profiles of B16F10 cells (WT), and B16F10 (NATURA) cells transfected with a scrambled siRNA (siRNA SCR), siRNA1A or siRNA1B. The reading was taken 48 h after transfection at 20 nM. MFIs for EGFP and tRFP are shown. (M) Flow cytometry-derived EGFP MFI normalized to tRFP MFI of two NATURA-expressing cell lines transfected with 20nM of siRNAs and collected at 48 h after transfection. Each dot represents an independent biological replicate. (N) NATURA-expressing B16F10 cells were transfected with GAPmer1 at various concentrations and analyzed through flow cytometry at 48 h after transfection. The three nucleotides at both 5′ and 3′ wings of GAPmer1 were modified with 2′OMe and full PS backbone. (O) Single-cell pPSI (FS) of the samples plotted in (N).

Three reporter proteins were selected for their proven applications in confocal microscopy and flow cytometry (i.e., turboRFP [tRFP] and EGFP) and *in vivo* imaging (i.e., firefly luciferase). The underlying approach to address the inherent variability in reporter expression is a gene structure that expresses the proteins that are each encoded as a spliced-isoform from a single gene construct. The NATURA reporter was implemented (and encoded in the plasmid named pC2.4) as a three-exon gene through which the expression of each protein is controlled by splice modulations (Figure 1A), described as follows.

Downstream an established ubiquitous promoter (EF1α), exon 1 of the NATURA gene codes for a nuclear localization signal to ease the automated fluorescence quantification of confocal images, followed by the sequence coding for the FLAG Tag.^20^ The subsequent gene module, encompassing intron 1, exon 2, and intron 2, was isolated and modified from the rabbit *C13H1orf43* gene’s exon 4 and its adjacent intronic regions, and was integrated proximal to the splice junctions. Exon 2, hereafter referred to as the frame shifter (FS), underwent targeted mutagenesis to reduce its length to 55 base pairs, resulting in a downstream mature mRNA with a reading frame of +2 upon splicing inclusion, or +1 when spliced out. *C13H1orf43* was selected as it is the orthologue of the ubiquitously expressed human C1orf43 (housekeepingHous gene),^21^ with exon 4 being consistently included in the splicing process. Furthermore, it is worth noting that adult C1orf43-orthologue 4933434E20Rik knockout mice (in heterozygosity) did not exhibit significant phenotypic alterations,^22^ thereby assuring that any potential sequence-dependent off-targeting of the NAT tool library on the orthologous mouse (and possibly human) gene would not culminate in adverse effects. Exon 3 of the NATURA gene was designed to possess two alternative acceptor splice sites (3′SS). The proximal 3′SS exon 3A that codes for tRFP and EGFP proteins in the +1 and +2 reading frames, respectively. The premature stop codons manifested in the tRFP sequence at the +2 frame were mutagenized into various amino acid codons. Analogously, the distal 3′SS, which is located downstream of the EGFP sequence and was cloned from a portion of the human *IL13RA1* intron, expresses exon 3B, which codes for luciferase protein in the +2 reading frame. Lastly, the rabbit β-globin polyadenylation (polyA) signal was appended. The final construct was inserted into a PiggyBac backbone for stable integration into the host genome in presence of a transposase. When stably integrated or transfected into various mouse and human cell lines, 84.5 ± 6.8% (by Taqman qPCR) (Figures S1A and S1B) of the expressed NATURA transcripts corresponds with the EGFP isoform, where exons 1, FS, and 3A are spliced-in and in-frame for EGFP (Figure 1A, top). The minor transcripts represent the tRFP isoform, which contains exons 1 and 3A (Figure 1B), and the Luciferase isoform, which contains exons 1, FS, and 3B (Figure 1C), constituting respectively, 8.6 ± 5.9% and 6.8 ± 3.2% (Figure S1B).

### NATURA tool library

SSOs, CRISPR-gRNAs, siRNAs, and GAPmers were designed to modulate the NATURA gene or (nascent or mature) transcripts. Multiple sequences for each mode of action (Table S1) were validated to enable sequence dependency investigation. The chemistries used for each NAT are listed in Table S1.

#### SSOs

Two groups of SSOs, labeled as arrangement-veering ASO (AVA) 1 and AVA2 were rationally designed to induce the exclusion of FS exon and exon 3A, respectively.^23^^,^^24^ AVA1 skips the FS exon and shifts the reading frame in the resultant mature transcripts from EGFP to the tRFP isoforms (Figure 1B), as confirmed by flow cytometry, agarose gel RT-PCR, western blot, and real-time confocal imaging (Figures 1D, 1E, and S1C–S1E). To account for the inherent variability in reporter expression, the efficiency of AVA1 was quantitated as FS’s FS exon percentage spliced-in (PSI). The PSI is calculated as the percentage of transcripts containing the FS exon over the total NATURA gene’s transcripts. This value can be calculated from the mRNA (as Taqman-derived PSI [tPSI]), or from the NATURA proteins quantification (named protein-derived PSI [pPSI]; see materials and methods). As expected, efficiency correlates inversely with both tPSI and pPSI. AVA1, uniformly modified with either 2′MOE or 2′OMe sugar moieties and full phosphorothioate (PS) backbone, was validated to be efficient in skipping the FS exon upon transfection in multiple cell lines, as confirmed by confocal imaging, in a dose-dependent manner (Figure S1F). The dose-dependent cellular toxicity profile of AVA1 was inferred from the cell number by concurrent analysis of nuclei count with high-content confocal imaging. Cellular toxicity was observed at concentrations above EC90, i.e., pPSI (EGFP) of <0.1 (lower dotted line, Figure S1F), of which the 2′OMe-modified AVA1 was more toxic than the 2′MOE. AVA1-induced switch was detectable upon free uptake, and was further enhanced under calcium chloride enriched medium^25^ (Figure S1G). The splice change was reversible (Figure S1H) and peaked at 24 to 48 h after transfection, depending on the cell type.

The SSO AVA2 was designed to block NATURA exon 3 proximal 3′SS (exon 3A) and force the use of the distal 3′SS (exon 3B), as validated by agarose gel and subsequent Sanger sequencing (Figures S1I and S1J), which converts the expression of EGFP to luciferase (Figure 1C) that can be measured upon luciferin addition to the medium (Figure S1K). The efficiency in inducing exon 3B-containing mature mRNAs, by 10 AVA2 candidates with varying potencies, is linearly correlated with luciferase protein luminescence intensity (Figure S1L). The NATURA transgenic mouse, containing the NATURA reporter inserted in the *Rosa26* locus, expressed a weak baseline luminescence in a dose-dependent fashion (Figure S1M). Upon a subcutaneous injection of AVA2, an increased luciferase emission in the peritoneal area was observed in the NATURA mice (Figure 1F). The luminescence was reversible and re-inducible upon a second AVA2 injection at 28 days after the first (Figure 1G).

#### CRISPR-Cas9 gRNA

Single gRNAs (sg1 and sg2) were designed to disrupt NATURA FS’s donor SS and exon 3A acceptor SS, respectively. The CRISPR-Cas9-mediated genomic introduction of InDels or substitutions within the respective splicing consensus sequences causes the switch between isoforms, while avoiding potential off-targets in both mouse and human genomes (Tables S2 and S3). Upon co-transfection of Cas9 protein and sg1 with different transfection reagents, or transduction with lentiCRISPRv2 viral particles containing the same gRNA sequence, cells showed a marked decrease in EGFP and a concomitant increase in the tRFP channel, indicating the loss of the FS exon 5′SS (Figures 1H, 1I, and S1N). Strikingly, 96% of the transduced cells lost the EGFP expression permanently after 5 days, while gaining a strong tRFP signal (Figure S1O). This is corroborated by the observation that the pPSI correlates linearly with the magnitude of the DNA lesions (Figures 1J, S1P, and S1Q). Further analysis of the linear correlation between tPSI and pPSI proved that the protein fluorescence readout is a reliable and reproducible substitute for cDNA quantification (Figure S1R). A similar trend of InDel efficiency across transfection reagents was observed in a different cell line albeit at a smaller magnitude, highlighting in turn the sensitivity of the system when using MFI quantification (Figure S1S). In contrast, sg2 led to increased luminescence in a luciferase assay upon CRISPR-Cas9 and gRNA transfection (Figure 1K) while decreasing both EGFP and tRFP signals (Figure S1T).

#### siRNA

Two non-overlapping siRNAs that target the FS exon were designed to degrade the dominantly expressed *EGFP* isoform. The residual but consistently present *tRFP* isoform (Figure S1B), which does not contain the FS exon and is thus not targeted by the siRNAs, is used as the normalizer to account for variability in gene expression. As evident by the fluorescence profiles (Figure 1L), while EGFP decreased upon siRNA transfection (top), the tRFP profile remained constant (bottom). In Figure 1M the EGFP protein knockdown efficiency by each siRNA is shown, normalized to tRFP on two cell lines. Alternatively, the normalized EGFP mRNA knockdown efficiencies can be obtained from the Taqman qPCR in which the NATURA’s FS exon-containing transcripts expression is normalized to a housekeeper gene (cyclophilin A) (Figure S1U). The knockdown efficiencies are similar when normalized to either tRFP protein or cyclophilin A transcripts. This is a consequence of both the siRNAs and the transfection reagent used not triggering differential transcriptional/translational alterations between the housekeeper and reporter genes. As a corollary to this, the internal normalization approach is advantageous for a knockdown mode of action given that the efficiency readout is not confounded by unspecific effects on the housekeeper gene expression or global transcription perturbations.

#### GAPmer

A complementary sequence targeting the FS exon was designed and synthesized as a GAPmer (Table S1). With the same principle of readout as the siRNA modality, transfection of the GAPmer resulted in a dose-dependent EGFP protein degradation (Figure 1N) but not tRFP (Figure S1V). Similarly, the degradation profile is dose-dependent and can then be internally normalized (Figure 1O) 48 h after transfection.

### Enhanced SSO potencies from specific MPCs of ribose chemistry

The NATURA system and the SSO tool library (AVA1s and AVA2s) were used to investigate effects of MPCs on an SSO’s potency and its cellular toxicity. Every ribose sugar in AVA1, labeled hereafter as AVA1A1, was applied with OML, or with 2′MOE and LNA moieties (MOL for short). To avoid confounding effects from considerable variations in melting temperature (T_m_), every mixmer contains three to four LNAs (15%–20% of the total length). An initial selection of 44 mixmers, each representing an MPC permutation were tested, with 22 in OML and 22 in MOL chemistry. For every mixmer and its mono-chemistry counterpart, the FS exon skipping efficiency (measured as pPSI) and the cellular toxicity (measured by cell count), were determined in NATURA-expressing cell lines. To quantitate the effects of an MPC on potency and cellular toxicity over the entire range of SSO concentrations, change in area under the curve (ΔAUC), defined as the cumulative difference of the AUC in the dose responses of pPSI or cell viability between a mixmer and untreated cells (see materials and methods), was determined.

**Figure 2:**
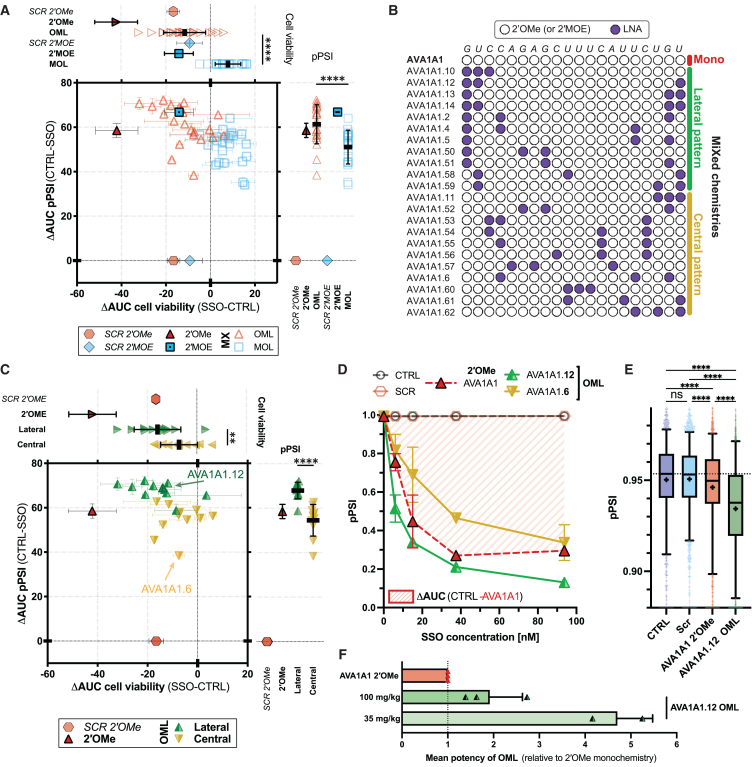
Positional configurations of SSO mixmers increase the potency of AVA1 (A) ΔAUC plot for 2′MOE, 2′OMe, MOL, and OML. Cumulative difference of the AUC in the dose responses between AVA1A1 SSO and untreated (CTRL) cells. For each SSO exon skipping efficiency (FS pPSI) and cellular toxicity (by nuclei count) over four concentrations (6, 15, 37.5, and 93.8 nM) were determined by high content confocal imaging at 24 h after transfection into CT26 NATURA-expressing cells. The AUC was calculated from the dose-response curve and subtracted with those from untreated cells to obtain the ΔAUC in pPSI (vertical axis) and cell viability (horizontal axis). (B) LNA placements (in purple) in AVA1A1 mixmers. (C) ΔAUC plot for AVA1A1 mixmers (OML), clustered by lateral and central PCs, and AVA1A1 (2′-OMe). Unpaired t test (Welch’s correction for unequal SD) was performed between lateral and central PC. Refer to Figure 2A legend. (D) pPSI dose-response curves of AVA1A1.12 (lateral MPC, OML), AVA1A1.6 (central MPC, OML), and AVA1A1 (2′OMe) obtained in CT26 NATURA cells at 24 h after transfection. ΔAUC of CTRL versus AVA1A1 in monochemistry is shaded for illustration purpose. (E) Single-cell flow cytometry- derived pPSI of ASGR^+^ dissociated cells from NATURA mice′s livers. Samples were collected and processed 48 h post-subcutaneous injection of SSOs (35 mg/kg). Kruskal-Wallis test with Dunn’s multiple comparisons (assuming non-Gaussian distribution). (F) Fold-change in pPSI change of OML mixmers compared with 2′OMe. Each datapoint represents an individual mouse.

In NATURA-expressing CT26 cells, both OML and MOL mixmers had lower cellular toxicities than their respective 2′OMe and 2′MOE oligos (Figure 2A, horizontal axis). The effect on potency was mixed (Figure 2A, vertical axis). Whereas all MOL mixmers were less efficient (i.e., lower ΔAUCs) than their 2′MOE counterparts, the relative efficiency of OML mixmers were either augmented or diminished compared with the 2′OMe AVA1A1. Among all features analyzed (% of GC containing LNA, LNA nucleotide compositions and combinations; data not shown and available upon request), only the relative LNA-substituted ribose positions within the mixmers could correlate with mixmers efficiencies. Specifically, AVA1A1 modified with lateral MPCs, which specify LNA placements at the 5′ first and/or second ribose (Figure 2B), have higher skipping efficiency (higher ΔAUCs) than mixmers modified with central MPCs whose LNA are placed elsewhere (Figure 2C). This and the following observations hold true for both OML and MOL mixmers (Figure S2A). While central MPC oligos tend to have higher T_m_s, no correlation with efficiency (either in pPSI and ΔAUC) was observed (Figures S2B–S2D). Although mixmers harboring either MPC patterns have similar cellular toxicity profiles, they are less toxic than the monochemistry counterparts. As an illustrative example, the pPSI dose-response curves of OML AVA1A1.12 (highest ΔAUC, lateral MPC), AVA1A1.6 OML (lowest ΔAUC, central MPC), and AVA1A1 (2′OMe) demonstrates the superior efficiency of AVA1A1.12 across the SSO concentrations consistently (Figure 2D), which was further confirmed from repeated transfection and mRNA quantification by Taqman qPCR (Figure S2E). Cell viability from AVA1A1.12 and AVA1A1.6 transfection was also improved over the monochemistry counterpart (Figure S2F).

The clustering feature of lateral and central MPCs was found to be conserved as the mixmers were screened on five more NATURA-expressing cell lines (B16F10, 3T3, HepG2, Huh7, and HeLa) (Figure S2G).

To validate the on-target activity of mixed chemistry oligos and to characterize the sensitivity of NATURA system *in vivo*, saline suspended mixmer AVA1A1.12 (OML) and AVA1A1 (2′OMe) were subcutaneously administered in NATURA mice with a single subcutaneous injection. The animals were sacrificed 48 h after injection and their livers dissected, dissociated, and stained for ASGR1, an hepatocytes marker. Consistent with cell culture observations, AVA1A1.12 OML mixmer outperformed the monochemistry oligo (Figure 2E). The splice-switching efficiency in hepatocytes was 4.7- and 1.9-fold higher for the lateral MPC oligo than its monochemistry counterpart at a low dose (35 mg/kg) and a high dose (100 mg/kg), respectively (Figures 2F, S2H, and S2I). In the higher dose, RNA was extracted from a portion of whole liver, and an average of 4.5-fold higher switch (tPSI) for the mixed chemistry than the monochemistry was detected by TaqMan PCR. The higher switch measured from tPSI in liver compared with pPSI in hepatocytes (Figure S2J) could be due to uptake from other cell types, the longer half-life of the EGFP protein compared with mRNA, or potential underestimation of the switching efficacy^26^ caused by autofluorescence in the GFP channel. Nevertheless, a correlation between pPSI in hepatocytes and tPSI for the whole liver was discerned (Figure S2K). Another experiment with NATURA transgenic mice was performed, using the highest concentration of SSOs and a longer evaluation endpoint (72 h). The results were confirmed in this setup in term of splicing switch in hepatocytes (Figures S2L and S2M). Part of the dissociated livers were sorted into ASGR^+^ and ASGR^−^ cells and Taqman PCR was performed, confirming the superiority of lateral MPC mixmer over monochemistry in both hepatocytes and other cells within the liver (Figure S2N).

Last, no acute hepatotoxicity or nephrotoxicity was observed at both doses and timepoints by probing for plasma and urine biomarkers (Figures S2O and S2P), nor significant caspase-3 activation in kidneys and livers (Figure S2Q).

**Figure 3:**
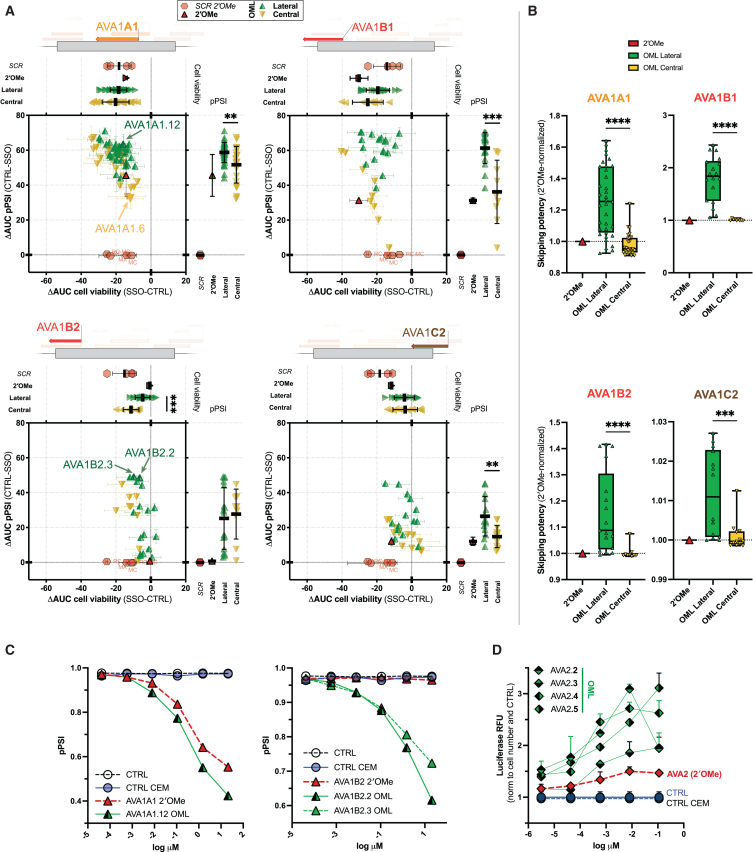
Mixmers positional rule applies to a large set of SSO sequences targeting the NATURA gene (Α) ΔAUC plot for AVA1A1, AVA1B1, AVA1B2 and AVA1C2 mixmers (OML), clustered by (refined) lateral and central MPC, and their respective monochemistries (2′OMe) from the secondary screen in CT26 NATURA cells. Controls used include a scrambled sequence with mono-chemistry (SCR.MC) and with three LNAs in a lateral PC (SCR.MX), complementary sequences to AVA1A1 (cAVA1A1, and cAVA1A1.12 with identical lateral MPC as AVA1A1.12), AVA1B1 (cAVA1B1), and AVA1C2 (cAVA1C2). (B) Relative skipping potency of AVA1s OML mixmers in free uptake with calcium-enriched medium (CEM). Huh7 NATURA cells were incubated with 118 nM of SSOs and analyzed by flow cytometry at 48 h. The potency (fold change) of OML mixmers over 2′OMe was obtained by normalizing the pPSI of the mixmer relative to CTRL with the pPSI of the monochemistry relative to CTRL. Each dot represents the average of a biological duplicate. Unpaired t test (Welch’s correction for unequal SD, one-tailed) between the lateral and central PC. (C) CEM dose-responses (pPSI) of representative OML mixmers and their respective monochemistries in Huh7 NATURA cells at 48 h after incubation. AVA1A1.12, AVA1B2.2, and AVA1B2.3 were modified with lateral MPC. Analysis performed through flow cytometry; each dot represents the median of at least 500 cells. (D) Luminescence of Huh7 cells at 48 h after incubation with AVA2 OML mixmers and monochemistry under CEM condition. The luciferase luminescence was first normalized to cell count which was detected using MTS assay and then normalized to the baseline intensity from untreated CEM condition. Mean and SEM of technical duplicates.

To test the MPCs’ adaptability on more sequences and base compositions, and to investigate MPCs over a range of substituted LNAs, three additional AVA1 (AVA1B1, AVA1B2, and AVA1C2) were rationally designed. Both AVA1B1 and AVA1B2 bind to FS exon’s 3′SS (acceptor), while AVA1C2 binds to the corresponding 5′SS (donor). Together with AVA1A1 (which bind to an ESE), the four SSOs were systematically modified with either lateral or central MPCs, with one to eight LNA substitutions as OML mixmers (Figure S3A). Their efficiencies and cellular toxicities were determined and analyzed as before, with the fluorescence and cell count measured by flow cytometry for increased throughput and sensitivity. With linear regression methods applied on the enlarged dataset (Figure S3B) (materials and methods), the mixed chemistry efficiencies are again differentiated only by LNA positions, while other parameters like SSO length, T_m_, GC%, number of LNA residues, or LNA base combinations are not associated with potency (data not shown and available through request). In detail, while LNA substitutions at the first three 5′ ribose remain the biggest contributor, substitutions at the last two or last three ribose are synergistic (Figure S3B). Thus, the clustering feature of lateral (refined) and central MPCs was corroborated in the secondary screen (Figures 3A and S3C), with the most efficient SSO for each AVA1 sequence modified with a lateral MPC. The only exception (out of 24 experimental conditions) is AVA1A1 in B16F10 cells, in which lateral MPC AVA1A1.10 is only the third most efficient. However, the top mixmers (ΔAUCs pPSI = 60.6 and 59.9), both with central MPC, are only marginally more efficient than AVA1A1.10 (ΔAUC pPSI = 56.6), but are considerably more toxic (ΔAUCs cell viability = −38.5 and −25.9 vs −12.2) (Figure S3C, first panel). Notably, lateral MPC oligos have superior efficiency than their monochemistry counterparts with up to 500-fold (Figure S3D, AVA1B2 in B16F12 cells). The results were further confirmed as the screen was repeated under free uptake in calcium chloride-enriched media (CEM) (Figure 3B). Cell viability not shown as no significant toxicity was observed for all the SSOs. Figure 3C plots the in-depth dose response of representative mixmers modified with lateral MPCs (AVA1A1.12, AVA1B2.2 and AVA1B2.3) and their respective monochemistry oligos, performed to validate the CEM screening. Notably, among mixmers modified with lateral MPCs, their relative order sorted by efficiencies was generally consistent between CEM and transfection conditions. On the other hand, no association between cell viability and number of substituted LNAs was discerned (Figure S3E).

The relative potency of a mixed chemistry was derived from the ratio of its ΔAUC(pPSI) to the monochemistry counterpart. The median potencies of OML mixmers modified with Lateral MPCs were always higher than mixmers modified with central MPCs (except for AVA1B2 in CT26 cells) and 2′OMe monochemistry oligos (Figure S3F). Their magnitudes however depend on the cell types and the SSO sequences—for instance, AVA1B2 mixmers recorded the highest relative potencies and the widest magnitude range (>18-fold) among cell lines. For each SSO, the optimal number of LNAs was inferred, in all cell lines tested, from mixmers with relative potencies within 10% from the most potent mixed chemistry. The respective optimal residues for AVA1A1, AVA1B1, AVA1B2, and AVA1C2 are three, five, five, and four LNAs (Table S4). This suggests the optimal LNA residues and the relative mixed chemistry potency are both inversely correlated with T_m_ of the natural SSO sequences—66°C, 57.84°C, 50.62°C, and 67.34°C, respectively. To test the hypothesis, AVA1A2, which overlaps AVA1A1 sequence but has the highest predicted T_m_ at 69.53°C, was designed and validated as before. None of the OML mixmers were more potent than the monochemistry counterpart except in Huh7 cells for which a paltry median relative potency of 1.1 was achieved with two substituted LNAs (Figure S3F; Table S4). This hints to a T_m_ threshold beyond which LNA substitution is futile. This could explain why MOL mixmers of AVA1A1 were less efficient than its 2′MOE counterpart in the initial screen (Figures 2A and S2A), given that T_m_ is raised by 0.9°C –1.7°C with each 2′MOE modification, which is higher than a 2′OMe nucleoside.^27^ Hence, a corollary is that for a given sequence, the optimal LNA residues for MOL mixmers will not be greater than in the corresponding OML mixmers. The secondary screen was subsequently repeated with MOL mixmers under CEM condition in Huh7 cells, and further validated the superior efficiency of MOL mixmers modified with lateral over central MPCs (Figure S3G). The optimal LNA residues inferred from AVA1B1 and AVA1B2 MOL mixmers are 4 and 3, respectively (Table S5), which are expectedly less than the corresponding OML mixmers. As also anticipated from the higher T_m_ of 2′MOE nucleosides, MOL mixmers of AVA1A1 were not more potent than its 2′MOE (median relative potency of mixmers with lateral MPC is 1.04) (Figure S3H), while mixmers of AVA1C2 managed to improve its ineffective monochemistry counterpart (Figure S3G; Table S5).

It is worth noting that all the top mixed chemistry performers in Lateral MPC did not elicit CCL22 expression in the BJAB assay^28^^,^^29^ (Figure S3I), nor significant Caspase-3/7 activation (Figure S3J) or CDKN1A upregulation^3^ (Figure S3K).

Last, AVA2 mixmers modified with lateral MPCs confirmed to induce higher EGFP-to-luciferase luminescence than their monochemistry counterpart (Figure 3D), hinting that the lateral MPC could be applied to SSOs targeting other exons other than NATURA’s FS exon.

**Figure 4:**
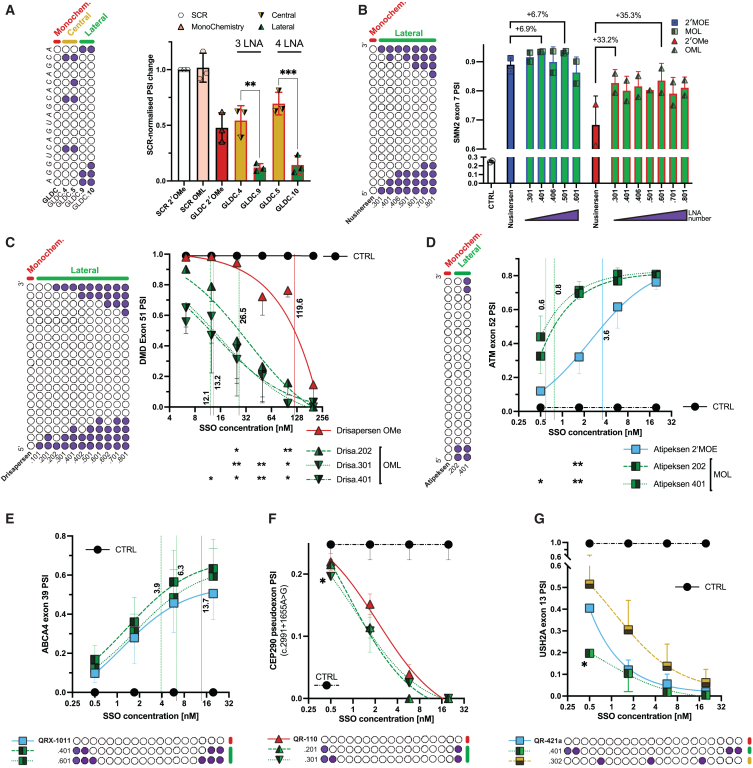
The mixmer improved positional configuration applies to SSO sequences targeting therapeutically relevant genes (A) Schematics of the mixmers modifications with either LNA (in purple) of the GLDC-targeting SSO. Relative change in PSI of exon 7 of GLDC transcripts upon treatment with SSOs at 100 nM in CEM for 72 h in A549 cells. Each dot represents a biological replicate. One-way ANOVA. (B) PSI of SMN2 exon 7 upon transfection with nusinersen (20 nM) and its mixmer permutations (in lateral MPC) for 24 h in SMN1 KO motoneurons. Percentage of improved inclusion of mixmers in comparison to monochemistry is shown above the bars. Each dot represents a biological replicate with a technical duplicate for each replicate. (C) PSI of DMD exon 51 upon transfection with drisapersen and its lateral MPCs in RH30 cells at 24 h. Biological duplicates with average of technical duplicates each. Nonlinear regression (three parameters) is fitted, statistical significance is shown for monochemistry vs. mixmers only. EC50 values are shown with colored vertical lines and calculated with 95% confidence interval. The same applies for all the other panels. (D) PSI of ATM c.7865C>T exon 51 minigene upon co-transfection with atipeksen and its lateral MPCs in 293T cells at 24 h. (E) PSI of ABCA4 c.5461-10T>C exon 39 minigene upon co-transfection with QRX-1011 and its Lateral MPCs in 293T cells at 24 h. (F) PSI of CEP290 pseudoexon (caused by the c.2991 + 1655A>G mutation) upon co-transfection of the minigene with QR-110 SSO and its lateral MPCs in 293T cells at 24 h. Statistical significance is shown only for SSO vs. Control (CTRL) (G) PSI of USH2A exon 13 minigene upon co-transfection with QR-421a and its lateral or central MPC in 293T cells at 24 h.

Lateral MPCs were then applied to seven SSO therapeutics targeting genes involved in human diseases. The first example shows how mixmers can simultaneously boost potency and reduces the molecular weight of an SSO that downregulates *GLDC* gene expression for anticancer therapy. The reported candidate is a 27-mer with full 2′OMe-PS modifications that induces exon 7 exclusion, resulting in frameshifted *GLDC* transcripts that are degraded by nonsense-mediated decay (NMD).^30^ The 5′ end was trimmed to a 17-mer, upon which lateral and central MPCs were applied and validated in A549 and Huh7 cells. Consistently, OML mixmers modified with lateral MPCs were more efficient than mixmers modified with central MPCs upon transfection and free uptake. The most potent mixed chemistry was notably more efficient than the published candidate (Figures 4A and S4A). In MOL composition, mixmers modified with lateral MPCs were again more efficient than 2′MOE and mixmers modified with central MPCs (Figure S4B). To explore the adaptability of *S*-cET sugar moiety to the MPCs, OML and MOL mixmers were converted to OMc (2′OMe+cET mixmer) and MOc (2′MOE+cET mixmer), respectively by substituting the LNAs within. In both OMc and MOc compositions, mixmers modified with Lateral MPCs were also observed to be more efficient than mixmers modified with central MPCs (Figure S4C).

The second example is nusinersen, a 2′MOE-PS SSO that facilitates SMN2 exon 7 inclusion and is an approved treatment for spinal muscular atrophy.^31^ Despite the nusinersen-mimic oligo has high efficacy—inducing exon 7 inclusion in nearly 90% of transcripts even at low doses in ALS patient-derived neurons—the most potent SSO resulted to be in MOL mixed chemistry, incorporating four substituted LNAs (Figure 4B; Table S6). When nusinersen sequence was synthesized as OML mixmers, the most potent SSO achieved up to a 35.3% improvement over the nusinersen sequence in 2′OMe SSO, although it is less efficient than the 2′MOE/MOL counterparts (Figure 4B). Marginal improvement in cell survival from MOL or OML mixmers were noted (Figure S4D).

A significant increase in efficiency was observed with the failed clinical candidate drisapersen, a 2′OMe-PS SSO that promotes dystrophin exon 51 exclusion as a treatment for Duchenne muscular dystrophy.^32^ The most potent OML Lateral MPC oligos, which did not affect cell viability (Figure S4E), demonstrated up to a 12.8-fold enhancement in exon skipping efficiency compared with drisapersen (Figure S4F; Table S7). These results were corroborated in a dose-response assay with EC50 going from 119.6nM for the monochemistry to 12.1nM for the most effective OML oligo (Figure 4C). This enhancement has also been validated *in vitro* and *in vivo* by another group,^18^ which reported that Lateral OML mixmers, particularly Drisa.202, achieved up to a 5-fold increase in exon 51 skipping in mouse heart tissues compared with the monochemistry oligo (AON-B8). Similarly, conversion of the drisapersen sequence to MOL mixmers showed a modest but consistent increase in efficiency for lateral MPCs relative to the drisapersen sequence in full 2′MOE monochemistry (Figure S4G).

Atipeksen is an SSO developed in an N-of-1 clinical context to correct the mis-splicing of ATM exon 53 in patients with c.7865C>T mutation.^33^ Strikingly, this full 2′MOE-PS oligo can be greatly improved by Lateral MPC MOL modification (5-fold improvement in EC50) (Figure 4D). In contrast, the same SSO with central MPC modifications underperformed relative to the 2′MOE Atipeksen, while all lateral MPC oligos outperformed it (Figure S4H, chemistry combinations and sequences are listed in Table S8).

Finally, we aimed to test the Lateral MPC in three eye-related therapeutic SSOs that failed their respective clinical trials for Stargardt disease (QRX-1011,^34^ targeting ABCA4 c.5461-10T>C mutation), Leber congenital amaurosis type 10 (QR-110,^35^ for CEP290 c.2991 + 1655A>G mutation), and Usher syndrome (QR-421a,^36^ directed against USH2A exon 13). The lateral MPC improvement over the 2′MOE QRX-1011 was more than 3-fold in EC50 (Figures 4E and S4H). Also for QR-110 sequence, the lateral MPC effectiveness was consistently better than the 2′OMe chemistry (Figures 4F and S4H). Finally, QR-421a sequence in the lateral MOL conformation halved the EC50 of its monochemistry counterpart (0.12 nM down from 0.31 nM), while being less effective in the central MPC (EC50 = 0.59 nM) (Figures 4G and S4H).

In conclusion, the study demonstrated that lateral MPC modifications were successfully applied to six NATURA SSOs (AVA1s and AVA2) and were the best performing chemistry across all the seven therapeutically relevant SSOs at all concentrations tested. Among these, four out of seven exhibited more than a 2-fold improvement with the adoption of Lateral MPC, with equal representation in MOL and OML mixed chemistries.

## Discussion

Several NAT-specific reporters have been published to evaluate their potency or delivery. NATURA is a multi-modality system enabling the evaluation of four classes of NATs, namely SSOs, GAPmers, siRNAs, and CRISPR-Cas9 systems, which allows rapid adaptation or comparison of a delivery system to different NATs. Besides addressing shortcomings from all prior labeled- and unlabeled-based approaches with its unique fluorescent and luminescent readouts for internally normalized functional NAT uptake on live cells or animals,^37^ the system supersedes tedious and eliminates potential PCR artifacts in RNA analyses while facilitating a seamless workflow from *in vitro* findings to *in vivo* validation. With both readouts validated to be sensitive to small changes in the relative abundance of the protein reporters, the system exhibits the ability for precise quantification of functional uptake. The NATURA system consists of a three-spliced-isoform reporter gene and an accompanying tool library containing multiple sequences for each of the four NAT classes. Besides enabling sequence (in)dependency investigations, the NAT sequences targeted in the library neither have mouse nor human origin unlike other reporter systems, and can avoid confounding the results arising from unintended targeting of the host endogenous genes. In a recent reporter designed for SSO modality, which also incorporated an SSO tool library, mKate2, and EGFP fluorescence proteins were co-expressed via a P2A ribosomal-skipping sequence, using the red-emitting protein as an housekeeper.^26^ The relative abundance of the proteins cannot however be universally used to account for inherent variability in the reporter expression among individual cells and across tissues, as P2A efficiency is not 100% and possibly cell-type dependent.

The use of mixed ribose chemistries concomitantly in an SSO has been attempted, with some studies reporting an improvement in SSO potency only in specific combinations or sequence.^14^^,^^15^^,^^16^^,^^17^^,^^19^^,^^37^ Recently, the Datson Lab reported four OML mixmers with improved potency compared with its 2′OMe monochemistry counterpart. They were modified with three MPCs that are defined in this study as “lateral MPC” and are present in AVA1A1.101, AVA1A1.203, and AVA1A1.12 (Figure S3A).^18^ Therefore, a systematic study to discover universal modification patterns that improve SSO potency, and to investigate their sequence (in)dependency, is warranted. Using NATURA and five sequences in its SSO tool library, general modification patterns, defined as lateral MPCs, were discovered for OML and MOL mixmers for which the most efficient mixed chemistry per SSO sequence was consistently found to be applied with lateral MPCs across all cell lines tested. Lateral MPCs superiority over monochemistry, although with different magnitudes due to the differences in splicing landscape, absorption, and pharmacodynamics, was validated *in vivo* at multiple concentrations and timepoints.

The finding was further corroborated on seven published SSO therapeutics; therefore, it is likely that the MPC is sequence agnostic as no specific base compositions was discerned. In the seven SSOs tested, the best performing chemistry has always been found to be the Lateral MPC over the monochemistry, at all concentrations tested. Moreover, the rule may be generalizable to mixmers using any combination of 2′-O-modified and constrained ribose including cETs. However, since the combination of ribose chemistries and the specific Lateral MPC that confer the highest SSO potency depend on the sequence and cell type, it is essential in drug discovery to screen for all possible lateral MPCs for each ribose chemistry combination. This becomes a practical approach given the drastically diminished mixmer permutations. In addition, depending on the cell types, lateral MPC mixmers were always observed to have either improved or identical cell survival as compared with their mono-chemically modified counterparts.

As defined in lateral MPCs, the placement of LNA substitutions at the flanks of an SSO with 5′ end more potent than at 3′ is likely to have a mechanistic basis, given that they are also agnostic to sequence, 2′-O-modification and cell type. When elucidated in future studies, the Lateral MPCs may apply to more dual or higher order combinations of ribose chemistries, and for which many chemical combinations have not been explored.

## Materials and methods

### Cell culture and transfection

The human embryonic kidney 293T (HEK293T), 3T3 (mouse embryonic fibroblasts), B16F10 (murine melanoma), Burkitt lymphoma B cell (BJAB), HeLa (human cervical carcinoma), HepG2 (human hepatocellular carcinoma), and Huh7 (human hepatocellular carcinoma) cell lines were procured from the American Type Culture Collection (ATCC). The CT26 (mouse colon carcinoma) and patient derived iPSCs (SMN1 knock-out) cell lines were kindly provided by Dr. Manikandan Lakshmanan and Dr. Shi Yan NG, respectively, of the Institute of Molecular and Cellular Biology in Singapore. These cell lines were propagated in accordance with ATCC guidelines.

To generate stable NATURA-expressing cell lines, 3 × 10^5^ cells were seeded in six-well plates containing 1 mL of complete growth medium the day prior to transfection. Transfection was carried out using 4.5 μg of NATURA plasmid (pC2.4) and 0.5 μg of PRP-mCherry-CAGhybase plasmid in conjunction with the Lipofectamine 3000 reagent after 16–20 h of incubation. The following day, the medium was replaced with complete growth medium supplemented with Blasticidine antibiotic (1:1,000, Sigma-Aldrich, cat- 15205) and the cells were grown under selection for 14 days. Subsequently, the cells were sorted based on EGFP expression.

The protocols outlined in our previous work^38^ were used for the viral packaging and transduction of the cells.

### Reverse transfection in 96-well plate

We combined 5 μL of ASO, at the required concentrations, with 20 μL of Optimem (#31985062; Thermo Fisher Scientific) in a 96-well cell culture treated plate. For each well, a mixture of 25 μL of Optimem and 0.9 μL of Lipofectamine RNAiMAX transfection reagent (#13778030; Thermo Fisher Scientific) was aliquoted. The lipid-ASO complex was allowed to form by incubating the plate for 20 min at room temperature on a gentle shaker (300 rpm). Meanwhile, cells were trypsinized, washed in PBS, and resuspended in complete growth medium at the final concentration of 20,000 cells/50 μL and 10,000 cells/50 μL of media for 24 h and 48 h of incubation, respectively. We then added 50 μL of the cell suspension to the ASO-RNAiMAX 96-well plate, shaken gently for 10 s at 300 rpm, and then placed in a humidified incubator. Cells were collected and processed further after 24 h, 48 h, and 72 h for SSO, GAPmers or siRNA, and CRISPR system analyses, respectively, unless stated otherwise.

### Agarose gel PCR and sequencing

For treatment of CT26-NATURA cell line with Scramble, AVA1 and AVA2 SSOs, 3 × 10^5^ cells were plated in 6-well plates with 1 mL of complete growth medium the day before transfection. After 16–20 h, or when the cells reached a confluency of 50%–60%, 100 nM of SSOs were transfected into cells using Lipofectamine RNAiMAX transfection reagent. The following day, RNA was extracted following the trizol protocol^39^ and 500 ng of RNA were reverse-transcribed into cDNA using Maxima kit (#K1642; Thermo Scientific). The required transcripts were amplified using Dreamtaq polymerase (#K1081; Thermo Scientific) and primers stated in Table S9. We used 2X DreamTaq Mastermix as a PCR mix in a total volume of 25 μL. Primers were used at the final concentration of 1 μM. All PCRs were performed at an initial holding temperature of 95°C for 3 min, 26–32 cycles of denaturation at 95°C for 45 s, annealing at 56°C for 30 s and elongation at 72°C for 40 s, and a final elongation temperature of 72°C for 3 min and 4°C holding temperature. PCR products were run in 1.7%–2.5% agarose gels and the product bands were visualized using ImageQuant RT ECL imager (GE Healthcare).

For DNA sanger sequencing, the PCR product was purified using the Favorgen PCR purification kit (#FAGCK 001; Favorgen) and sequenced using primers stated in Table S9.

### High content imaging

AVA1 SSO candidates were screened by reverse transfecting 4 NATURA expressing cell lines (CT26, B16F10, 3T3, and Huh7) at four different concentrations: 6 nM, 15 nM, 37.5 nM, and 93.75 nM. After 24 h from transfection, cells were first fixed using 4% PFA and then stained with Hoechst 33342 stain (1:3000) (#H3570; Invitrogen) for 15 min at room temperature. Images were captured with Opera Phenix high content screening system and analyzed with Columbus software. Average and SD of two biological replicates are shown in the graphs. A minimum of 6 fields and 250 cells were imaged for each replicate. Unpaired t test was performed between OML and MOL (Welch′s correction, unequal SDs, one-tailed *p* value). The same pipeline was used for the analysis of all samples. Raw image files and pipeline files can be provided upon request.

### FACS analysis

Cells were trypsinized and washed in PBS. Collected cells were resuspended in ice-cold 5% FBS/PBS and analyzed within 1hr using the Becton & Dickinson LSRII or Penteon-Novocyte Agilent L5 analyzer. The HTS module was used for analyzing 96-well plates.

The laser settings for the EGFP and tRFP channels were kept consistent throughout the experiments and across different cell lines. For experiments requiring absolute count, events/sec were recorded while keeping the volume of sample constant.

pPSI and cell count was obtain from at least 500 cells for each replicate. Acquisition was performed 24 h after transfection. Each datapoint represents the average and SD of two independent biological replicates. Unpaired, nonparametric Mann-Whitney test (one-tailed) was performed between lateral and central PCs.

### Luciferase assay

Live cells were stained by adding 0.4% trypan blue (1:1, #15250061; Gibco) and counted using Countess II FL automated cell counter (Thermo Fisher Scientific). Luciferase assay (#E1960; Promega) was performed by lysing 1 × 10^5^ cells in a flat white 96-well plate with 22 μL of 1× passive lysis buffer at room temperature for 15 min. Subsequently, the sample was incubated with 100 μL of luciferase assay substrate for 5 min in humidified incubator. Readings were taken with Tecan multimode spark 10M plate reader.

### MTS assay

Cells were trypsinized and resuspended in 80 μL of complete growth medium with 16 μL of CellTiter 96 AQueous One Solution Reagent (#G3580; Promega). Absorbance was measured at 490 nm after 1 h of incubation in a humidified incubator at 37°C using Tecan multimode spark 10M plate reader.

### DNA fragment length capillary electrophoresis (genescan)

Genomic DNA was extracted from cells with Qiagen DNeasy kit (#69506; Qiagen) as per the manufacturer’s instructions. Further genescan analyses were performed using the protocol described in our previous publication.^38^

### SSO innate immunity activation assay in BJAB cells

In a 96-well plate, 0.5 × 10^6^ BJAB cells were incubated with 2 μM of SSO in 100 μL of cell culture medium for 24 h. The next day, RNA was extracted following the trizol protocol,^39^ and 500 ng of RNA were reverse-transcribed into cDNA using the Maxima kit (#K1642; Thermo Scientific). The cDNA obtained was then analyzed using an SYBR green qPCR Assay (#A25742; Applied biosciences) with primers and probes specified in (Table S9).

Each reaction mix consisted of 10 μL of 2× SYBR green master mix, 2 μL of primer mix (10 μM forward and reverse primers), and 8 μL of cDNA (3.33 ng/μL). The qPCR assays were performed on a Bio-Rad CFX96 Real-time system using the following cycling conditions: 10 min at 95°C, followed by 40 cycles of 15 s at 95°C, and 1 min at 55°C. The data were analyzed using GraphPad Prism 9. ISIS104838 and ISIS353512 were used as negative and positive controls, respectively.^29^

### Differentiation of motor neurons

On day 0 of the differentiation process, 1 × 10^6^ pluripotent iPSCs were placed in a standard 10 cm dish pre-coated with Matrigel, using iPS brew (#130-107-086; Miltenyi Biotec) with 5 μM ROCK inhibitor Y-27632. The next day, the media was switched to Neural Induction Media (NIM), composed of a basal mixture (50% DMEM/F12 (#11320-033; Gibco), 50% Neurobasal media (#130-093-570; Miltenyi Biotec), 1x GlutaMAX (#35050-061; Gibco), 1× non-essential amino acids (#11140-050; Gibco), 1x N2 (#17502048; Gibco), 1x Neurobrew (#130-093-566, Miltenyi Biotec)), and supplemented with 0.5 μM LDN193189 (#72147; Stem Cell Tech) and 4.25 μM CHIR99021 (#130-104-172; Miltenyi Biotec). Starting from day 3, NIM was further enriched with 1 μM retinoic acid (RA; R2625; Sigma). Motor neuron progenitor cells were expanded from Day 10 onward in motor neuron progenitor expansion medium, which included basal medium supplemented with 1 μM RA and 1 μM purmorphamine (#130-104-465; Miltenyi Biotec). Beginning on day 17, the cells were cultured in motor neuron maturation medium, which contained basal medium supplemented with 10 ng/mL GDNF (#130-129-546; Miltenyi Biotec), 10 ng/mL BDNF (#130-103-435; Miltenyi Biotec), and 200 μM ascorbic acid (#A4544; Sigma). The motor neurons became ready for use in experiments starting from day 25.

### Western blot

Harvested cell culture pellets were resuspended in RIPA protein lysis buffer (150 nM sodium chloride, 50 mM Tris pH 7.5, 1% NP40) and incubated on ice for 10 min. Cells were then sonicated using Bioruptor UCD-200 sonicator for three pulses, 45s/15s on/off cycle at medium amplitude before spinning down at 20,000×*g* for 10 min at 4°C. The supernatant was collected, and protein was quantified using RC DC protein assay (#5000122; Bio-Rad) following the manufacturers protocol. Equal amounts of protein (40–50 μg) were run on 4%–20% SDS-PAGE gel and transferred to a PVDF membrane (#IPVH00010; Immobilin-p). Subsequently, the membrane was blocked (5% milk, #1706404; Bio-Rad), probed with primary (overnight, 4°C) and secondary (1–2 h, room temperature) antibody, and developed using SuperSignal West Pico (#34577; Thermo Fisher Scientific) or Femto chemiluminescent substrate (#34080; Thermo Fisher Scientific). The images were captured using Bio-Rad ChemiDoc reader. Antibodies are listed in Table S10.

### Taqman assay

The cDNA obtained as described in the Agarose gel and PCR section was analyzed by Taqman Assay (#4304437; Thermo Fisher Scientific) using primers and probes specified in Table S9.

Each reaction mix contained 10 μL of 2× Taqman master mix, 1 μL of NATURA primer-probe mix (2.5 μM probes and 10 μM primers), 1 μL of Cyclophilin A primer-probe mix (2.5 μM probes and 10 μM primers) and 8 μL of cDNA (3.33 ng/μL). The assays were run on Bio-Rad CFX96 Real time system using cycling conditions: 2 min at 95°C followed by 40 cycles of 5 s at 95°C and 40 s at 60°C with no dissociation curve. The data was analyzed using GraphPad prism 9.

### Animal studies and NATURA mouse generation

The NATURA mouse (C57BL/6 strain) was generated by Cyagen Biosciences by insertion of the NATURA gene in the Rosa26 locus. The gRNA to mouse ROSA26 gene (CTCCAGTCTTTCTAGAAGATGGG), the donor vector pNATURA (EF-1α promoter-sequence, tRFP-EGFP-luciferase module, rBG polyA cassette), and Cas9 mRNA were co-injected into fertilized mouse eggs to generate targeted knockin offspring. F0 founder animals were identified by PCR followed by sequence analysis, which were bred to wildtype mice to test germline transmission and F1 animal generation.

All animal work complies with regulations established and approved by the Institutional Animal Care and Use Committee (IACUC #R21-1350 and #221727) in accordance with Republic of Singapore’s guidelines.

### SSO treatment of NATURA mice

Isofluorine-anesthetized NATURA mice were treated with SSOs: Scramble (NC2/SCR), AVA1 (35 mg/kg or 100 mg/kg) and AVA2 (25 mg/kg) via subcutaneous injections. The SSOs were resuspended in sterile saline solution and injected according to the IACUC protocol.

### *In vivo* luciferase assay

The luminescent response of NATURA mice treated with AVA2 was assessed using the IVIS Spectra apparatus at various time intervals following SSO injection. For imaging purposes, the mice received a subcutaneous injection of 150 mg/kg luciferin (#P1041, Promega) and were subsequently imaged starting from 2 min after injection, with a 1-s exposure time.

### Organ preparation for FACS

Freshly harvested liver samples were first sliced and then centrifuged at 300×*g* for 3 min at 4°C to remove the supernatant. The remaining sample was resuspended in 5 mL of dissociation medium (containing 100× collagenase, 10× Dispase II, and 100× DNase I in PBS) and gently shaken at 37°C for 90 min in the dark. After dissociation, the sample was homogenized with an 18G needle, filtered through a 70 μm strainer, and collected by centrifugation at 200×*g* for 3 min.

The sample was then subjected to red blood cell lysis by incubating it in 5 mL of ACK buffer for 5 min at room temperature. Then, 5 mL of ice-cold PBS was added, followed by centrifugation at 150×*g* for 5 min at 4°C. This process was repeated with an additional 10 mL of ice-cold PBS. The sample was finally resuspended in 400 μL of ice-cold PBS and divided into two wells of a V-bottom 96-well plate for antibody staining: 1 well served as an unstained control, and the other was stained for hepatocytes and Kupffer cells.

After centrifuging the plate at 300×*g* for 3 min at 4°C and discarding the supernatant, the stained cells were resuspended in 100 μL of Live/Dead stain (1:1000 in PBS; #65-0868-14; Life Technologies) for 10 min at room temperature in the dark. The unstained cells were resuspended in 100 μL of PBS. Both sets of cells were then topped up with 100 μL of PBS, centrifuged at 300×*g* for 3 min at 4°C, and washed.

The stained sample was further incubated with a primary antibody for ASGR1 (1:50 in staining buffer; #11739-1-AP; Protein tech) for 45 min on ice in the dark, while the unstained sample was resuspended and incubated in staining buffer. After incubation, the stained set of samples were washed and then probed with anti-rabbit A647 secondary antibody (1:1,000; # A31573 Invitrogen) and anti-F4/80 rat monoclonal antibody (1:50; #123131; Biolegend) in 50 μL of staining buffer for 45 min on ice in the dark. The unstained sample remained in staining buffer. Finally, after another wash with staining buffer, the samples were resuspended in ice-cold PBS for flow analysis.

### pPSI and tPSI calculation

tPSI measures the fraction of FS exon-containing mature transcripts, which is derived by qPCR by calculating the abundance of the TaqMan probe spanning the FS exon to exon 3A (PR2-3A) (Figure S1B) over the sum of all the probes (PR2-3A + PR2-3B + PR1-3A) (Figure S1B). Protein-derived PSI (pPSI) measures the fraction of EGFP protein translated from the NATURA gene. It is obtained from the MFIs of EGFP and tRFP proteins:

(Equation 1) pPSI=MFI(EGFP)MFI(EGFP)+MFI(tRFP)

In case the value is calculated for single cells, the MFI is substituted with the actual fluorescence value of the EGFP and tRFP of each individual cell.

#### *In vivo* toxicity studies

Renal toxicity markers, KIM1 (#OKBB00352; Aviva Systems Biology) and creatinine (#E2CT-100; Enzychrom) were measured in mouse urine samples, while B2M (#OKEH0034; Aviva Systems Biology) was measured in plasma samples following the manufacturers protocol. The urine samples were diluted to 1:100 and plasma to 1:10,000 before use.

Mouse liver and kidney samples were stained and analyzed in immunohistochemistry by GLP-certified Advanced Molecular Pathology Laboratory (AMPL) for hematoxylin and eosin and cleaved caspase-3 staining with cleaved caspase-3 (Asp175) (5A1E) rabbit monoclonal antibody (Cell Signaling Technologies, #9664).

#### *In vitro* toxicity studies

Apoptosis was induced by irradiating the cells with UV light at 500 mJ/cm^2^ for 1 min following the protocol of Nijhawan et al.^40^ Subsequently, the cells were collected at 16hrs time point for further analysis.

Caspase activation was measured using the Caspase-Clo 3/7 kit (#G8091; Promega) following the manufacturers protocol. The readings were normalized against cell count detected using the MTS Assay (#G3582, Promega).

#### Minigenes cloning and SSOs co-transfection

The minigenes were built with the help of SpliceAI^41^ and cloned into pRP backbones by VectorBuilder. The plasmids and their maps are available on Addgene (ID:226553, 226554, 226555, and 22656). The minigenes (1 μg) and SSOs at specified concentrations were co-transfected into 293T cells in 12-well plates using Lipofectamine 3000 (#L3000001; Invitrogen), following the manufacturer’s protocol. Samples were collected 24 h after transfection for further analysis.

#### Linear regression model and MPC score calculation

A numerical score was assigned to a mixed chemistry based on the presence of LNA substituted at the first, second, and third 5′ positions (pF1, pF2, and pF3, respectively), and at the last, second last, and third last 3′ positions (pL1, pL2, and pL3, respectively) (see Figure S3B). pMid denotes the number of LNA substitutions in between the first three and last three positions. The MPC score below correlated with the mixed chemistry efficiency. By assigning the lateral pattern to oligos with the positional score higher than 5 we can see a significant clustering of the best performing oligos in this category (Figures 3A, S3A, and S3B).

(Equation 2) MPCscore=(7.3539∗pF1)+(8.7594∗pF2)+(−2.1391∗pF3)+(−0.6627∗pMid)+(1.4602∗pL3)+(−0.4381∗pL2)+(2.4611∗pL1)+(pF1∗pF2∗−7.1709)+(pF1∗pF3∗−2.4764)+(pF2∗pF3∗−6.6527)+(pL3∗pL2∗0.2970)+(pL3∗pL1∗−3.9413)+(pL2∗pL1∗3.4685)+(pF1∗pF2∗pF3∗11.9806)+(pL3∗pL2∗pL1∗−2.7352)

#### Statistical analysis

The statistical analysis for each figure is listed in the figure legend. All the statistical analysis were performed with GraphPad Prism Software (Version 10.2).

## Data and code availability

The authors confirm that the data supporting the findings of this study are available within the article and its Supplementary materials. Negative correlation analysis and raw data are available upon request. The pC2.4 plasmid, coding for the NATURA gene, as well as the plasmids coding for the ABCA4, CEP290, and USH2A minigenes are available on Addgene (plasmid ID: 226557 for pC2.4, other IDs are listed in Table S11).

## Acknowledgments

We would like to thank past and present members of the Wee lab for providing input and discussions. We acknowledge A∗STAR Biological Resource Center for their efforts in monitoring and care of the mice used in this study. This work was funded by the Industry Alignment Fund - Pre-positioning Program (H20C6a0034) to T.T. and K.B.W., and the Cell and Gene Therapy GAP Fund (ACCL/19-GAP009-R20H) to K.B.W. J.R.O. is supported by the A∗STAR Career Development Fund (C210812001). T.T., E.G., and K.B.W. are co-inventors in a patent filing relating to NATURA.

## Author contributions

T.T. conceived the NATURA system and designed its constructs. T.T., D.W., and E.G. planned the experiments. T.T., T.A., J.R.O., P.N., H.K., P.Y.Q.S planned and carried out the experiments. S.Y.N. isolated and provided the SMN1 KO iPSCs. X.L.G. performed the high content imaging confocal acquisition. T.T., W.Y.C., and T.A. analyzed the data. Statistical analysis and linear regression modeling was performed by W.Y.C. T.A., R.S.R.G., J.R.O., M.L., and H.L. carried out the animal studies. D.W. designed the GAPmers and SSOs. W.Y.C. designed the minigenes. T.T., D.W., and E.G. took the lead in writing the manuscript. All authors provided critical feedback and helped shape the research, analysis and manuscript.

## Declaration of interests

T.T., D.W., and E.G. are co-founders of Immunoa Pte. Ltd. A patent application for the NATURA and the chemical positional rules for SSOs have been filed by T.T., D.W., J.R.O., and E.G.

## Supplemental information

Data S1. Tables S1–S11

Document S2. Article plus supplemental information
