# Supplementary material for: Unveiling sequence-agnostic mixed-chemical modification patterns for splice-switching oligonucleotides using the NATURA platform
Source: Mol Ther Nucleic Acids. 2025 Jan 7;36(1):102422. doi: 10.1016/j.omtn.2024.102422 (PMC11803158; doi:10.1016/j.omtn.2024.102422)
Supplement: Document S1. Figures S1–S4 [file mmc1.pdf]

OMTN, Volume *36*

Supplemental information

Unveiling sequence-agnostic mixed-chemical modification patterns for splice-switching oligonucleotides using the NATURA platform

Tommaso Tabaglio, Taniya Agarwal, Wei Yuan Cher, Jin Rong Ow, Ah Keng Chew, Priscila Yun Qian Sun, Raja Sekhar Reddy Gurrampati, Hongfang Lu, Praveena Naidu, Hong Kai Ng, Xavier Le Guezennec, Shi Yan Ng, Manikandan Lakshmanan, Ernesto Guccione, and Keng Boon Wee

Figure S1

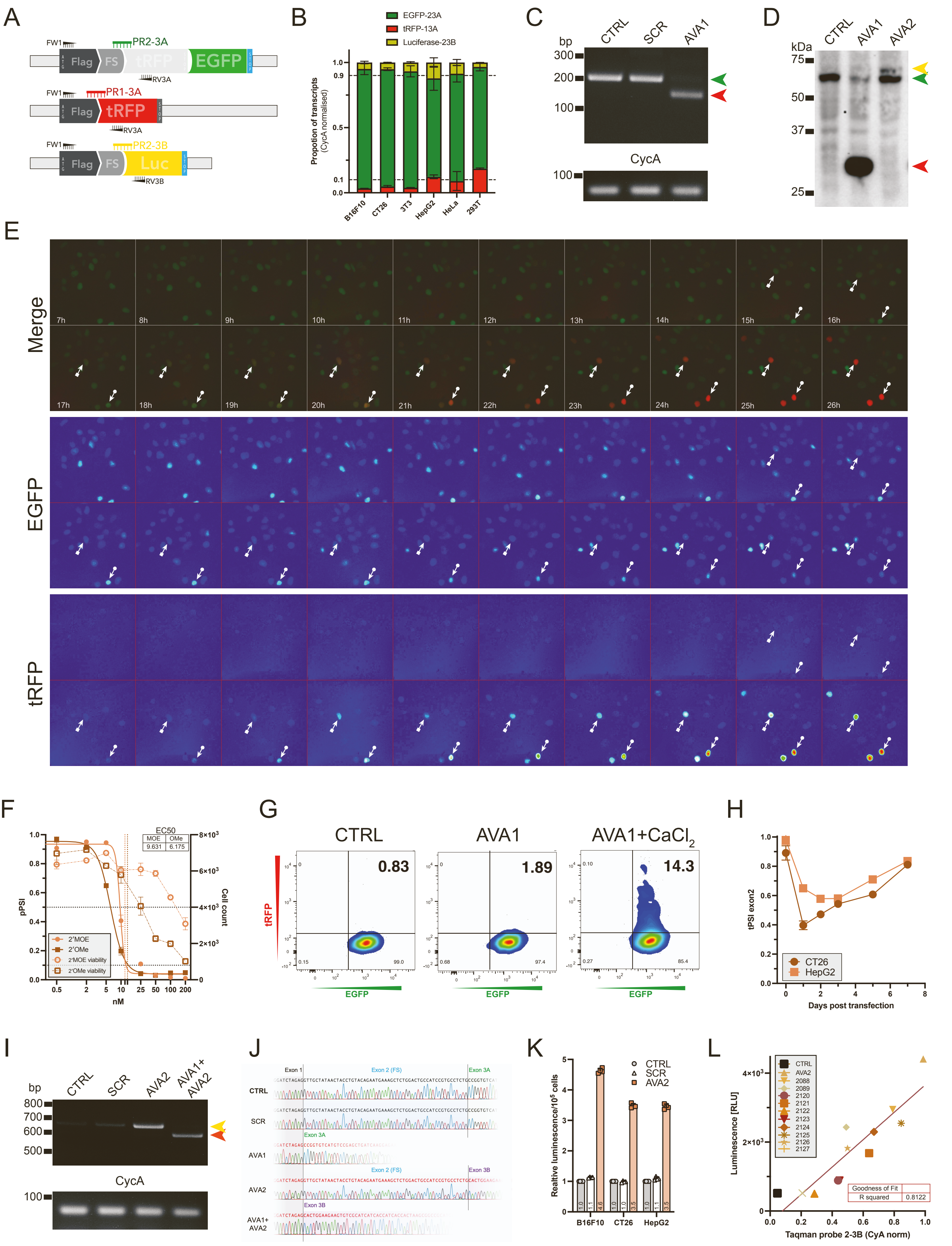
A
FW1
PR2-3A
Flag
FS
tRFP
EGFP
RV3A
PR1-3A
Flag
tRFP
RV3A
PR2-3B
Flag
FS
Luc
RV3B
B
EGFP-23A
tRFP-13A
Luciferase-23B
Propotion of transcripts (CycA normalised)
1.0
0.9
0.5
0.1
0.0
B16F10
CT26
3T3
HepG2
HeLa
293T
C
bp
CTRL
SCR
AVA1
300
200
100
CycA
100
D
kDa
CTRL
AVA1
AVA2
75
50
37
25
E
Merge
7h
8h
9h
10h
11h
12h
13h
14h
15h
16h
17h
18h
19h
20h
21h
22h
23h
24h
25h
26h
EGFP
tRFP
F
EC50
MOE
OMe
9.631
6.175
pPSI
Cell count
2'MOE
2'OMe
2'MOE viability
2'OMe viability
nM
G
CTRL
AVA1
AVA1+CaCl2
0.83
1.89
14.3
tRFP
EGFP
H
tPSI exon2
CT26
HepG2
Days post transfection
I
bp
CTRL
SCR
AVA2
AVA1+ AVA2
800
700
600
500
CycA
100
J
Exon 1
Exon 2 (FS)
Exon 3A
CTRL
SCR
AVA1
AVA2
Exon 3B
AVA1+ AVA2
K
Realtive luminescence/10^5 cells
CTRL
SCR
AVA2
B16F10
CT26
HepG2
L
CTRL
AVA2
2088
2089
2120
2121
2122
2123
2124
2125
2126
2127
Luminescence [RLU]
Goodness of Fit
R squared
0.8122
Taqman probe 2-3B (CyA norm)

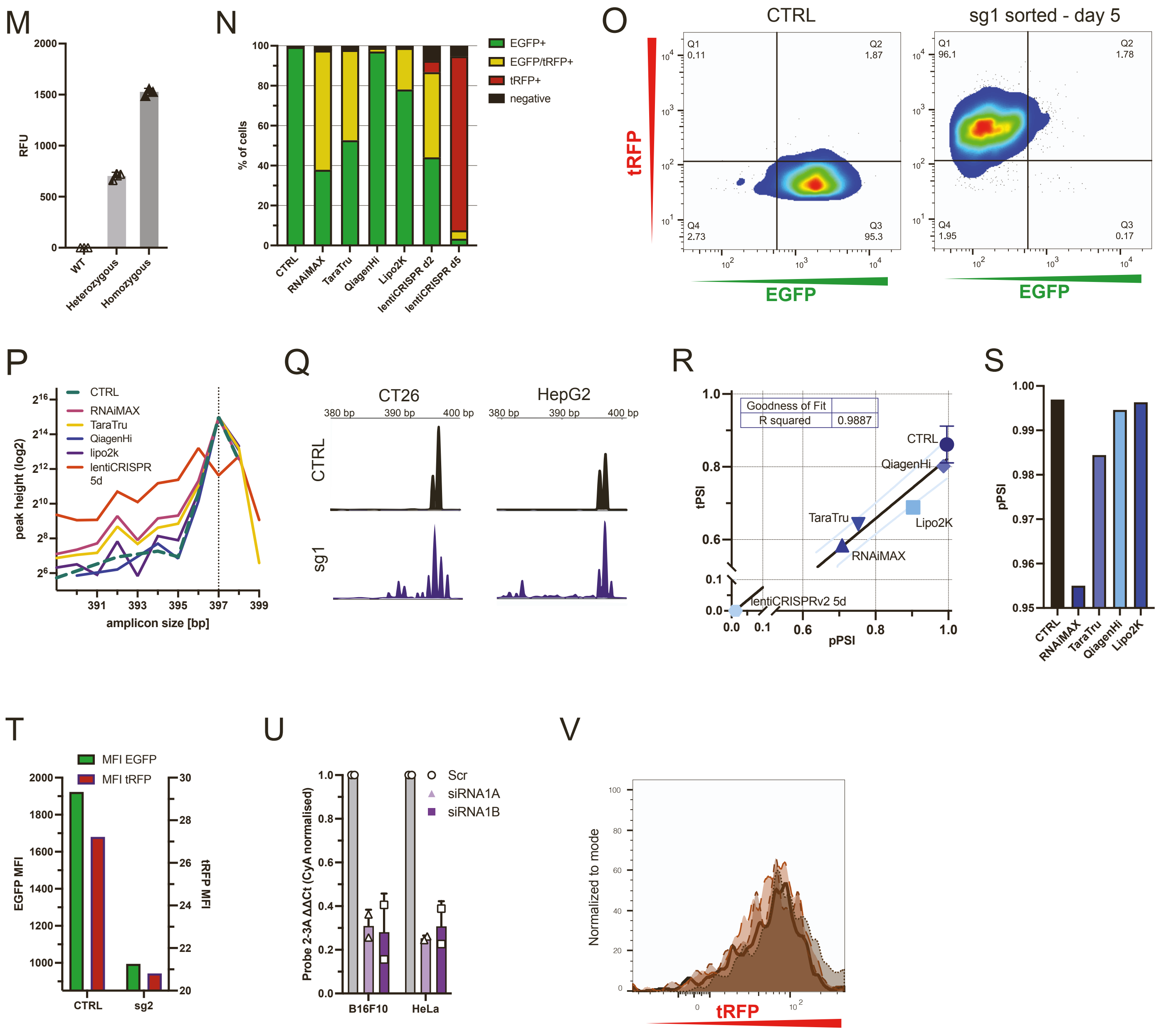
M
N
O
P
Q
R
S
T
U
V
RFU
WT
Heterozygous
Homozygous
% of cells
EGFP+
EGFP/tRFP+
tRFP+
negative
CTRL
RNAiMAX
TaraTru
QiagenHi
Lipo2K
lentiCRISPR d2
lentiCRISPR d5
CTRL
sg1 sorted - day 5
tRFP
EGFP
Q1 0.11
Q2 1.87
Q4 2.73
Q3 95.3
Q1 96.1
Q2 1.78
Q4 1.95
Q3 0.17
peak height (log2)
amplicon size [bp]
CTRL
RNAiMAX
TaraTru
QiagenHi
lipo2k
lentiCRISPR 5d
CT26
HepG2
380 bp
390 bp
400 bp
CTRL
sg1
Goodness of Fit
R squared 0.9887
tPSI
pPSI
CTRL
QiagenHi
TaraTru
Lipo2K
RNAiMAX
lentiCRISPRv2 5d
pPSI
MFI EGFP
MFI tRFP
EGFP MFI
tRFP MFI
CTRL
sg2
Scr
siRNA1A
siRNA1B
Probe 2-3A ΔΔCt (CyA normalised)
B16F10
HeLa
Normalized to mode
tRFP

FIGURE S1

A. TaqMan qPCR primers and probes scheme for the NATURA gene.
B. TaqMan qPCR values from different cell lines transfected with the NATURA plasmid.
C. Agarose gel of NATURA amplicons obtained from CT26 cells transfected with either a scrambled or AVA1 SSO. Cyclophilin A housekeeper was used as a loading control.
D. Western blot of protein lysates obtained from NATURA-expressing CT26 cells transfected with 100nM of a scrambled, AVA1 SSO, or AVA2 SSO, probed for FLAG-tag antibody. Cells were collected at 48h post-transfection.
E. Real time confocal imaging of NATURA-expressing CT26 cells transfected with 100nM of AVA1.
F. Flow cytometry-derived pPSI and cell count of 3T3 cells at 24 hours after transfection with AVA1 uniformly modified with either 2'O-MOE or 2'OMe sugar moieties with full phosphorothioate (PS) backbone.
G. Flow cytometry of NATURA-expressing HepG2 cells incubated for 48h with 1 μM of AVA1 (2'OMe, central panel) and in presence of 9mM calcium-enriched media (CEM, $CaCl_2$, right panel).
H. Time-course of tPSI (FS), calculated from TaqMan qPCR data, from two cell lines, CT26 and HepG2, after a single 10nM transfection of AVA1 with RNAiMAX.
I. Agarose gel and subsequent Sanger sequencing of NATURA amplicons obtained from CT26 cells transfected with a scrambled SSO, AVA2, or AVA1 and AVA2. Cyclophilin A housekeeper was used as a loading control.
J. Sanger sequencing of the splicing products after transfection with AVA1, AVA2, or AVA1 and AVA2 SSOs.
K. Untreated-normalized (CTRL) luminescence of NATURA-expressing cell lines; B16F10, CT26 and HepG2; treated with 100nM of scramble (SCR) and AVA2 SSO for 48 hours.
L. Linear correlation between Cyclophilin A-normalized TaqMan probe 2-3B, which measures abundance of Exon 3B-containing NATURA mature transcripts, and luminescence. NATURA-expressing HeLa cells were transfected with 50nM of each of the ten AVA2 candidates separately, and 15,000 cells were collected for luciferin incubation at 24 hours post-transfection.
M. Relative Fluorescence Units of the peritoneal mouse area for wild-type C57BL/6 mice, and NATURA heterozygous and homozygous mice after luciferin injection. ROI of the same size were drawn over the shaved region of the mice. The luciferase signal was measured as average radiance (photons/s/sr/cm2).
N. Relative abundance of each spliced isoform of the NATURA gene in CT26 cells transfected with lentiCRISPRv2 sg1 using different commercial transfection reagents.
O. NATURA-expressing CT26 cells were transduced with lentiCRISPRv2-sg1, sorted for EGFP-/tRFP+ and left in culture for 5 days prior to analysis through flow cytometry.
P. Capillary electrophoresis analysis of MPCR products surrounding FS splice donor site for the samples described in Fig. 1J. The height of the peak represents the abundance of a specific transcript.
Q. Capillary electrophoresis peaks of FAM-labelled MPCR products surrounding FS donor splice site (5'SS) in CT26 and HepG2 cells transfected with lentiCRISPRv2 sg1.
R. Correlation between pPSI (EGFP) and taqman-derived tPSI (FS) of the samples described in Fig. 1J.
S. pPSI of FS measured in NATURA-expressing B16F10 cells transfected with ALT+R Cas9/tracrRNA/sg1 using different commercial transfection reagents.
T. MFIs of EGFP and tRFP from NATURA-expressing B16F10 cells, transduced with lentiCRISPRv2-sg2.
U. TaqMan qPCR on the same samples described in Figure 1M.
V. Flow cytometry values of tRFP for the experiment described in Fig. 1O.

Figure S2

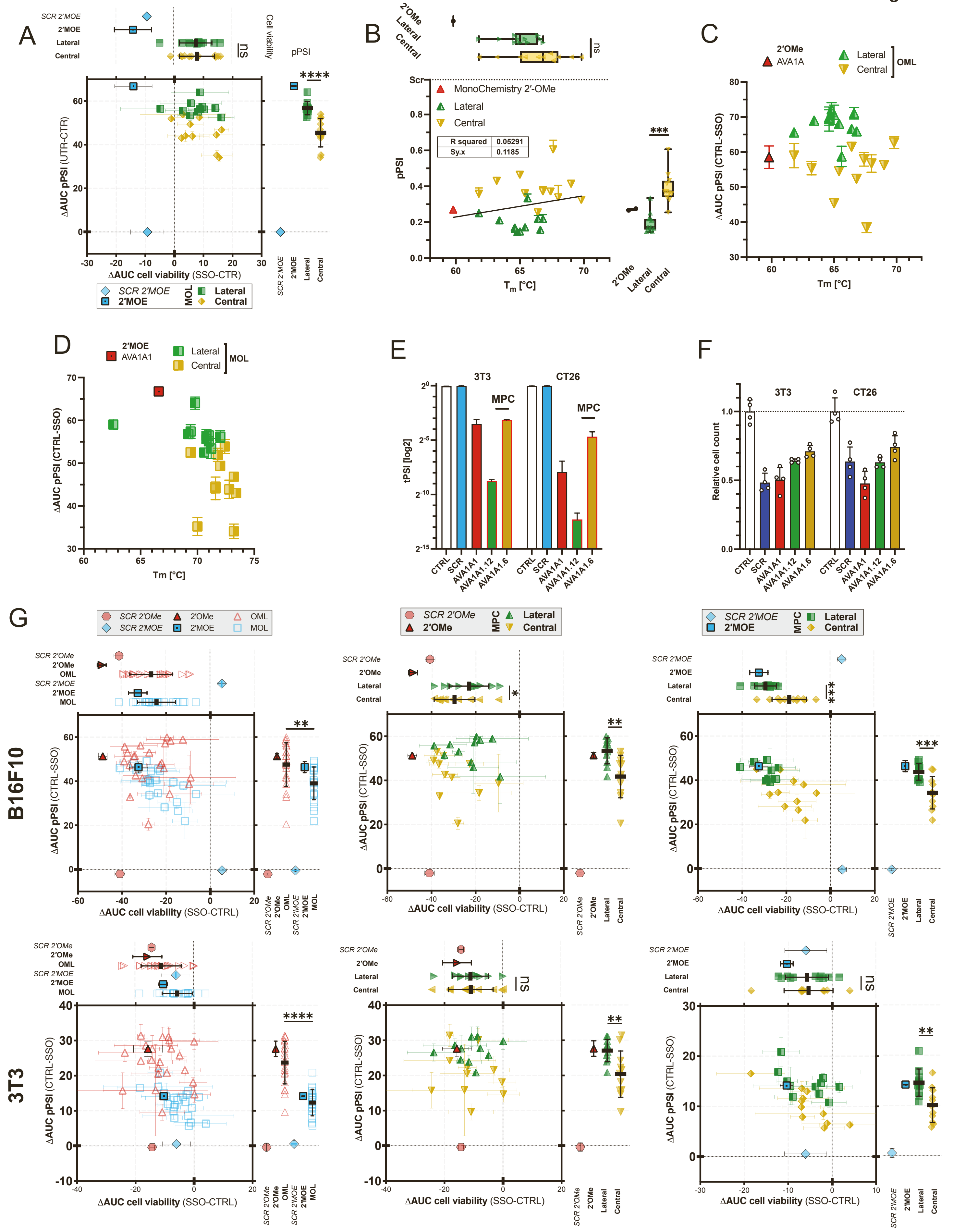
A
SCR 2'MOE
2'MOE
Lateral
Central
ns
Cell viability
pPSI
****
ΔAUC pPSI (UTR-CTR)
ΔAUC cell viability (SSO-CTR)
MOL
B
2'OMe
Lateral
Central
Scr
MonoChemistry 2'-OMe
R squared 0.05291
Sy.x 0.1185
pPSI
Tm [°C]
***
C
2'OMe
AVA1A
OML
ΔAUC pPSI (CTRL-SSO)
Tm [°C]
D
2'MOE
AVA1A1
E
3T3
CT26
MPC
tPSI [log2]
CTRL
SCR
AVA1A1
AVA1A1.12
AVA1A1.6
F
Relative cell count
G
B16F10
3T3
SCR 2'OMe
SCR 2'MOE
ΔAUC cell viability (SSO-CTRL)
**
*

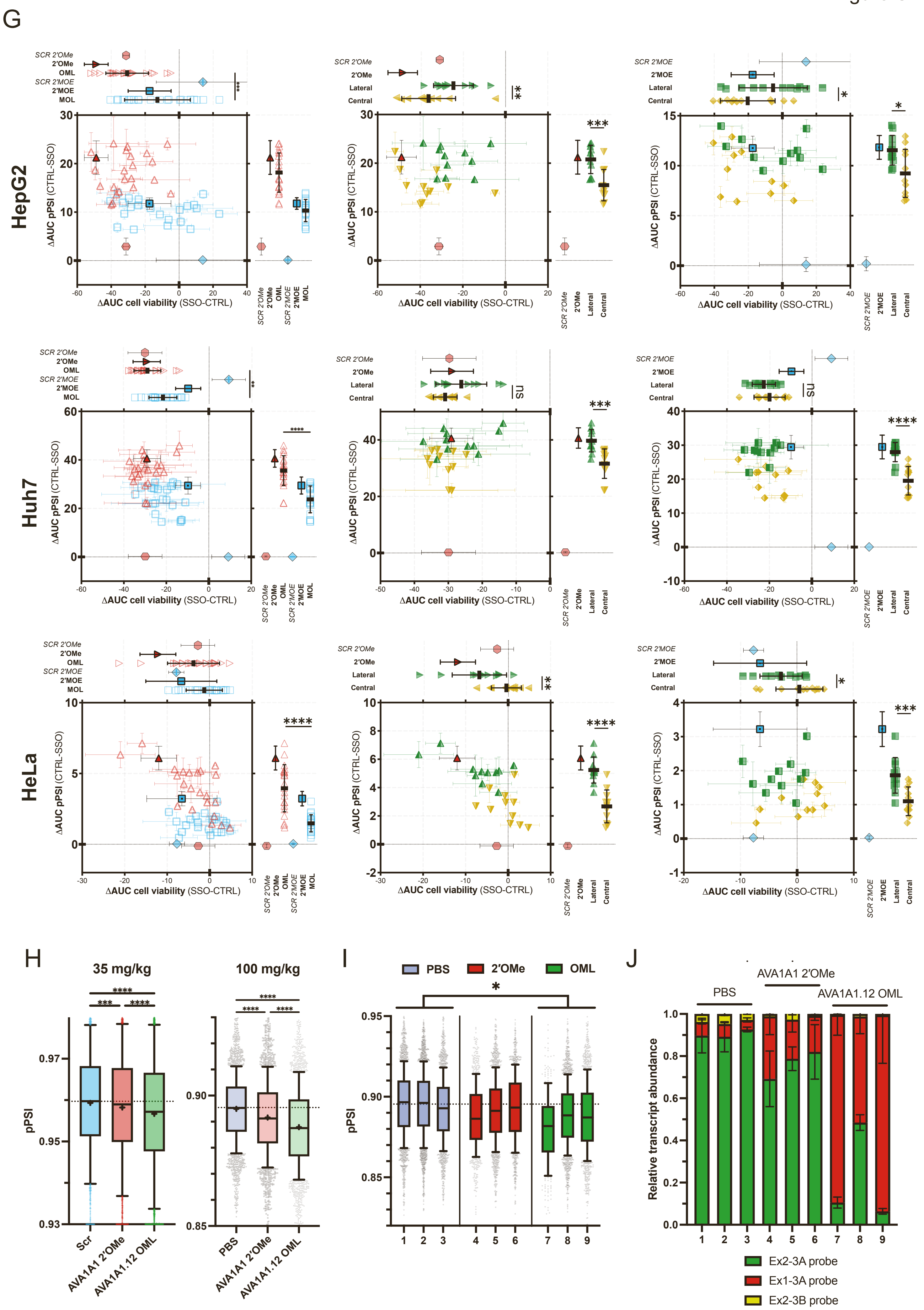
G
HepG2
Huh7
HeLa
SCR 2'OMe
2'OMe
OML
SCR 2'MOE
2'MOE
MOL
Lateral
Central
ΔAUC pPSI (CTRL-SSO)
ΔAUC cell viability (SSO-CTRL)
ns
H
35 mg/kg
100 mg/kg
pPSI
Scr
AVA1A1 2'OMe
AVA1A1.12 OML
PBS
I
PBS
2'OMe
OML
J
PBS
AVA1A1 2'OMe
AVA1A1.12 OML
Relative transcript abundance
Ex2-3A probe
Ex1-3A probe
Ex2-3B probe

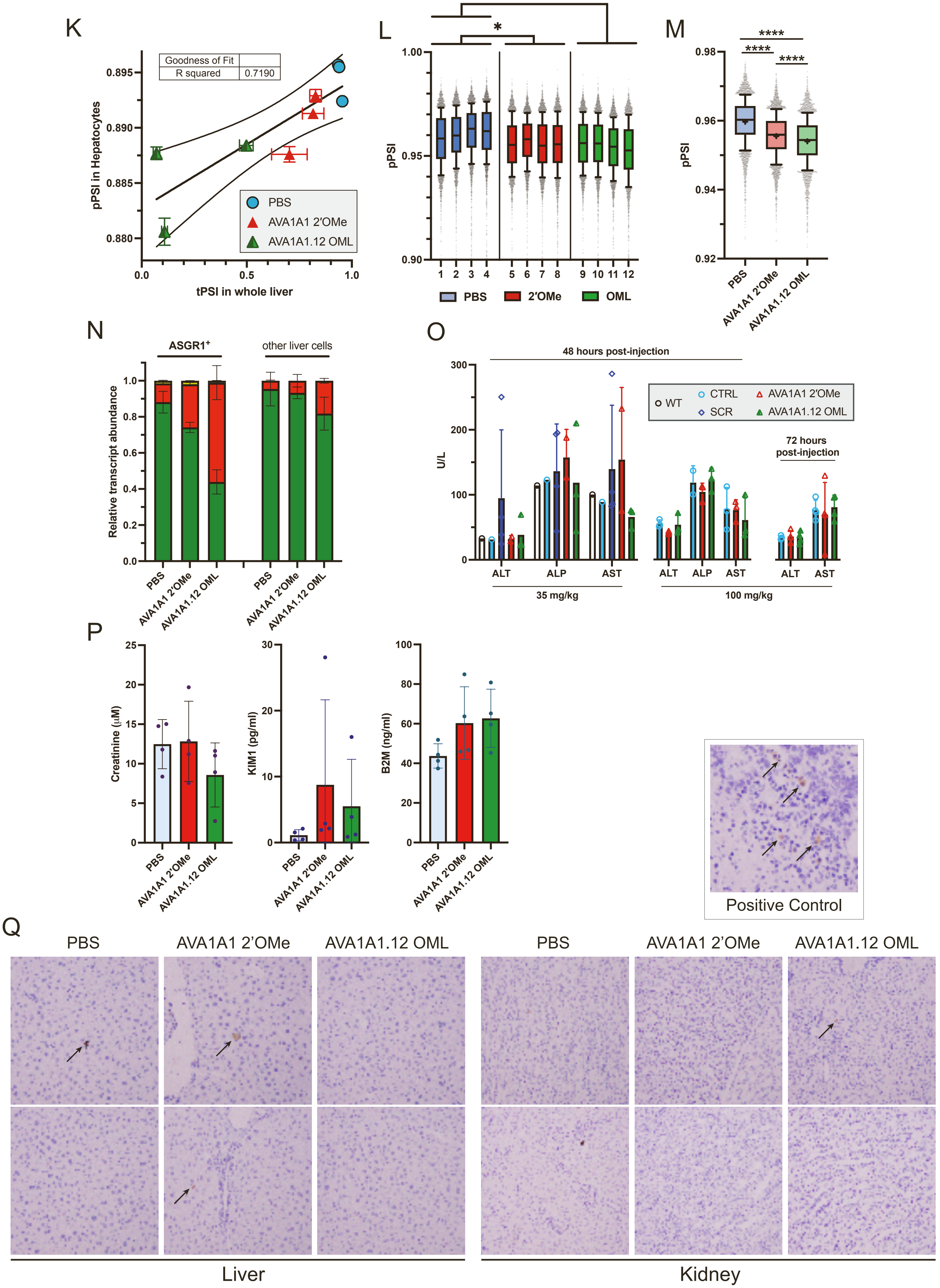
K
Goodness of Fit
R squared 0.7190
pPSI in Hepatocytes
tPSI in whole liver
PBS
AVA1A1 2'OMe
AVA1A1.12 OML
L
pPSI
PBS
2'OMe
OML
M
pPSI
PBS
AVA1A1 2'OMe
AVA1A1.12 OML
N
ASGR1+
other liver cells
Relative transcript abundance
PBS
AVA1A1 2'OMe
AVA1A1.12 OML
O
48 hours post-injection
72 hours post-injection
U/L
WT
CTRL
SCR
AVA1A1 2'OMe
AVA1A1.12 OML
ALT
ALP
AST
35 mg/kg
100 mg/kg
P
Creatinine (μM)
KIM1 (pg/ml)
B2M (ng/ml)
PBS
AVA1A1 2'OMe
AVA1A1.12 OML
Positive Control
Q
PBS
AVA1A1 2'OMe
AVA1A1.12 OML
Liver
Kidney

FIGURE S2

A. ΔAUC plot for AVA1A1 mixmers (MOL), clustered by Lateral and Central MPCs, and AVA1A1 monochemistry (2'MOE). Transfection in CT26-NATURA cells. Refer to Fig. 2A legend.
B. pPSI of AVA1A1 mixmers transfected at 37.5nM in CT26 cells, clustered by Lateral and Central MPCs, plotted against their experimental melting temperature (Tm).
C. ΔAUC of pPSI of AVA1A1 OML mixmers and 2'OMe in CT26 cells, clustered by Lateral and Central MPCs, plotted against their experimental melting temperature (Tm).
D. ΔAUC of pPSI of AVA1A1 MOL mixmers and 2'MOE in CT26 cells, clustered by Lateral and Central MPCs, plotted against their experimental melting temperature (Tm).
E. TaqMan-derived PSI (tPSI) of AVA1A1.12 (Lateral MPC, OML), AVA1A1.6 (Central MPC, OML) and AVA1A1 monochemistry (2'OMe) obtained from 3T3 and CT26 NATURA cells at 24hour post-transfection at 37.5nM. Each bar represents a technical triplicate.
F. Manual cell count, normalized for Untreated (CTRL), of the corresponding cells analyzed in panel E. Each dot represents an replicate measurement.
G. ΔAUC plot for AVA1A1 mixmers (OML and MOL) and monochemistries (2'OMe and 2'MOE) obtained from B16F10, 3T3, HepG2, Huh7, and HeLa NATURA-expressing cell lines. Left panel: refer to Fig. 2A legend. Middle and right panels: ΔAUC plots clustered by Lateral and Central MPCs for AVA1A1 mixmers (OML) and AVA1A1 mixmers (MOL) respectively. Refer to panel 2C for the statistical analysis.
H. Biological replicate of the experiment described in panel 2E. Left panel: Each box represents a single mouse. Right panel: 3 mice receiving the same treatment (PBS, AVA1A1 2'OMe, AVA1A1.12 OML) are merged into a single box plot. Ordinary one-way ANOVA with Turkey's correction applied.
I. pPSI derived from flow cytometry of ASGR+ cells as described in figure 2E. Each box plot represents an individual mouse, while each dot represents a single cell. Nested ANOVA.
J. Relative abundance of transcripts coding for EGFP (Ex2-3A), tRFP (Ex1-3A), and Luciferase (Ex2-3B) obtained from whole liver cDNA and subsequent TaqMan MPCR as summarized in figure S1A.
K. Correlation between tPSI (obtained from the values in figure S2J) and pPSI (obtained from values in figure S2I).
L. Single-cell pPSI derived from flow cytometry of ASGR+ cells of mice injected with 100mg/kg SSOs or PBS control (in blue) at 72h post-treatment. Each box plot represents an individual mouse, while each dot represents a single cell. Nested ANOVA.
M. pPSI of the cells from the experiment described in panel L, grouped by treatment. One-way ANOVA.
N. TaqMan MPCR for the ASGR1+- and ASGR1--sorted cells from the liver of mice treated with 100mg/kg for 72h. In Green, the relative abundance of the PR2-3A probe (EGFP), in Red the PR1-3A probe.
O. Acute liver toxicity markers (ALT, ALP, AST) from blood of mice used in all the in vivo experiments described previously. Blood was collected at 48 hours post injections. Each dot represents a single mouse.
P. Acute renal toxicity markers ELISA values for mice treated with 100mg/kg at 72h. Each dot represents a mouse. The average of two technical replicates is shown.
Q. Immunohistochemistry liver and kidney slides stained for active Caspase-3 in mice treated as described in panel P. Two representative fields are shown for each treatment. The positive control for the staining is a mouse tumor sample where Caspase-3 has been proven to be active.

Figure S3

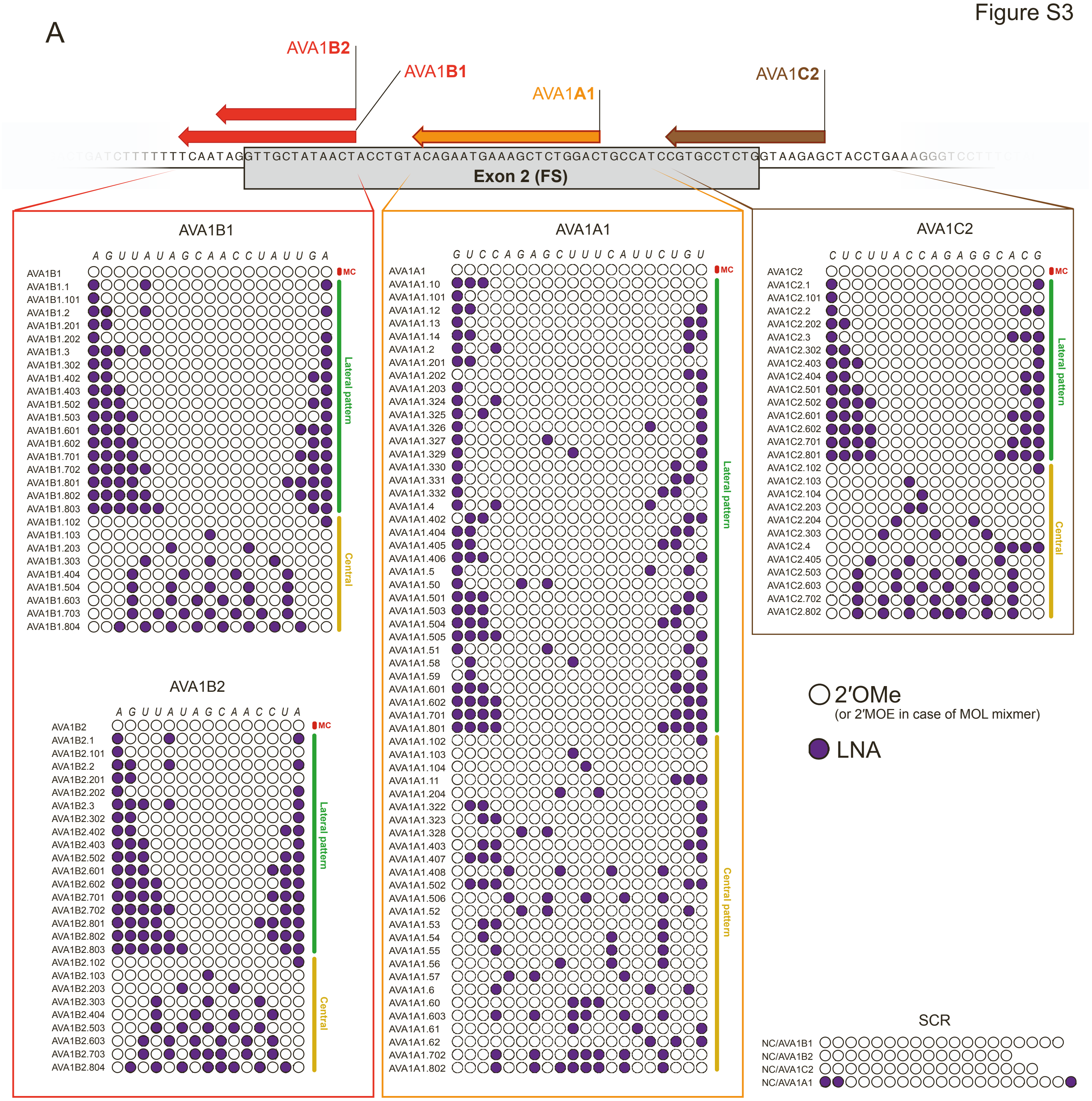

B

Predictors	mean_auc_delta Estimates	CI	p
(Intercept)	22.98012	16.24974 – 29.71049	**<0.001**
pF1	7.35421	3.72806 – 10.98036	**<0.001**
pF2	8.76283	0.67604 – 16.84962	**0.034**
pF3	-2.14070	-7.36779 – 3.08640	0.421
pMid	-0.66213	-1.48669 – 0.16244	0.115
pL3	1.46175	-3.81880 – 6.74230	0.587
pL2	-0.43896	-6.16580 – 5.28788	0.880
pL1	2.46236	-0.93035 – 5.85506	0.154
pF1 × pF2	-7.17159	-16.45529 – 2.11211	0.130
pF1 × pF3	-2.47808	-16.18001 – 11.22384	0.722
pF2 × pF3	-6.65764	-18.07215 – 4.75687	0.252
pL3 × pL2	0.29255	-10.13145 – 10.71656	0.956
pL3 × pL1	-3.94726	-13.80495 – 5.91042	0.432
pL2 × pL1	3.46908	-3.35723 – 10.29539	0.318
(pF1 × pF2) × pF3	11.98871	-5.77652 – 29.75394	0.185
(pL3 × pL2) × pL1	-2.72699	-16.41252 – 10.95855	0.695

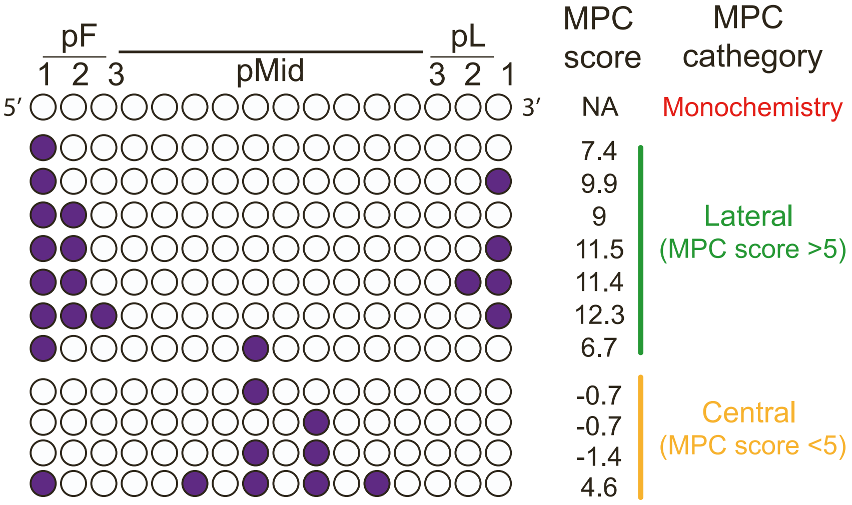

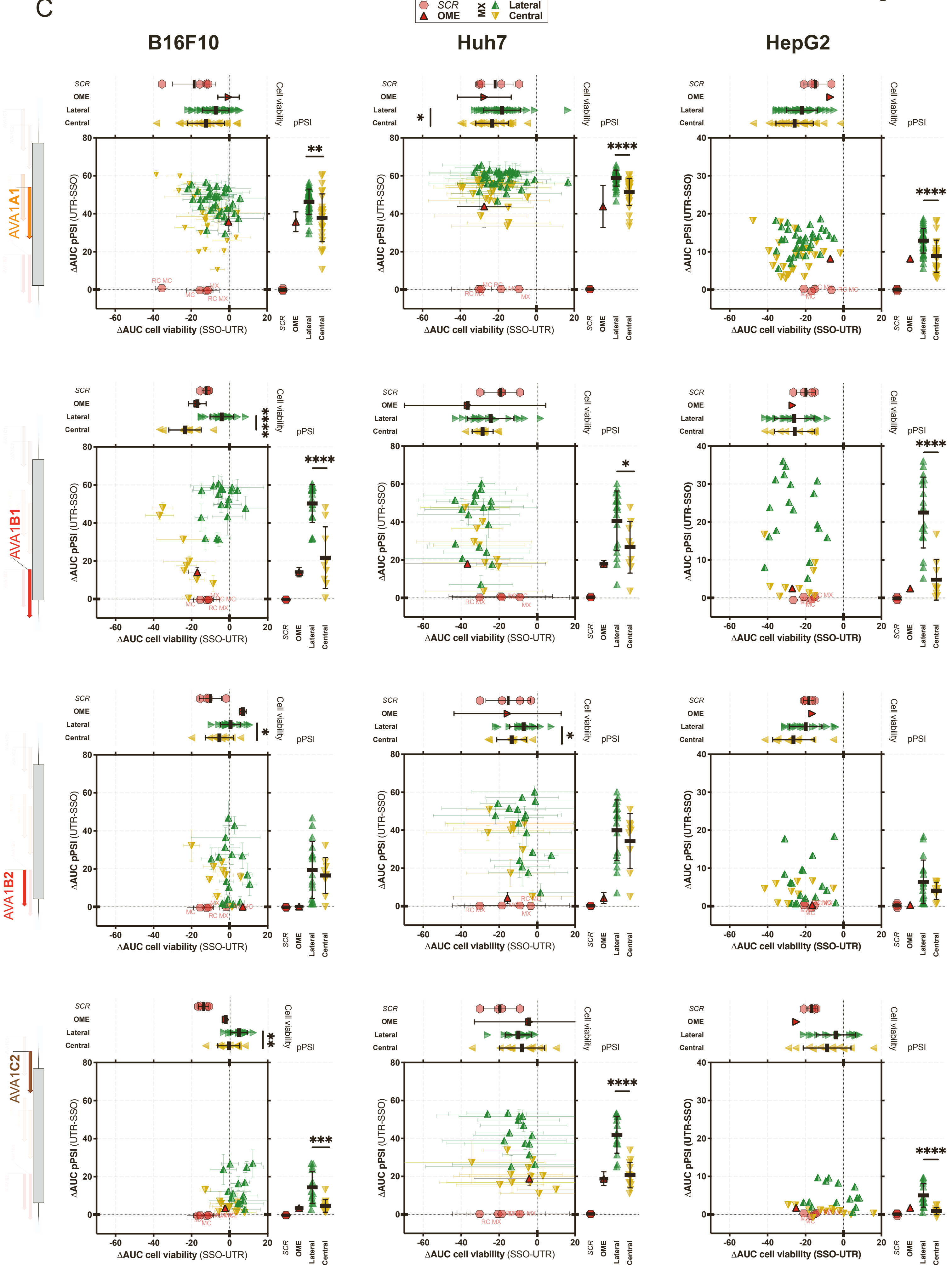
C
SCR
OME
MX
Lateral
Central
B16F10
Huh7
HepG2
AVA1A1
AVA1B1
AVA1B2
AVA1C2
Cell viability
pPSI
ΔAUC pPSI (UTR-SSO)
ΔAUC cell viability (SSO-UTR)
RC MC
RC MX
MC
MX
**
****
*
***

Figure S3

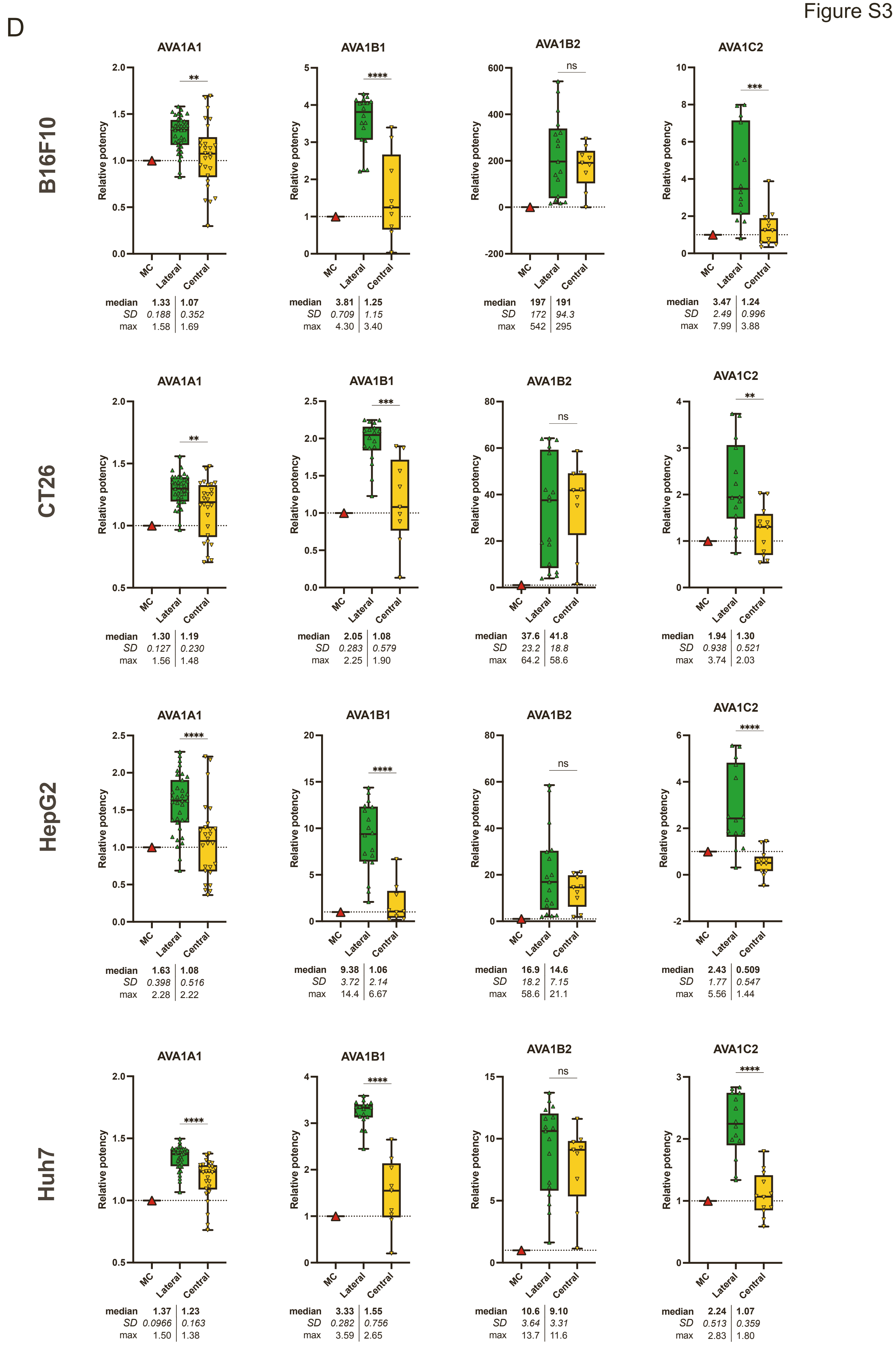
D
B16F10
AVA1A1
Relative potency
**
MC Lateral Central
median 1.33 | 1.07
SD 0.188 | 0.352
max 1.58 | 1.69
AVA1B1
****
median 3.81 | 1.25
SD 0.709 | 1.15
max 4.30 | 3.40
AVA1B2
ns
median 197 | 191
SD 172 | 94.3
max 542 | 295
AVA1C2
***
median 3.47 | 1.24
SD 2.49 | 0.996
max 7.99 | 3.88
CT26
AVA1A1
**
median 1.30 | 1.19
SD 0.127 | 0.230
max 1.56 | 1.48
AVA1B1
***
median 2.05 | 1.08
SD 0.283 | 0.579
max 2.25 | 1.90
AVA1B2
ns
median 37.6 | 41.8
SD 23.2 | 18.8
max 64.2 | 58.6
AVA1C2
**
median 1.94 | 1.30
SD 0.938 | 0.521
max 3.74 | 2.03
HepG2
AVA1A1
****
median 1.63 | 1.08
SD 0.398 | 0.516
max 2.28 | 2.22
AVA1B1
****
median 9.38 | 1.06
SD 3.72 | 2.14
max 14.4 | 6.67
AVA1B2
ns
median 16.9 | 14.6
SD 18.2 | 7.15
max 58.6 | 21.1
AVA1C2
****
median 2.43 | 0.509
SD 1.77 | 0.547
max 5.56 | 1.44
Huh7
AVA1A1
****
median 1.37 | 1.23
SD 0.0966 | 0.163
max 1.50 | 1.38
AVA1B1
****
median 3.33 | 1.55
SD 0.282 | 0.756
max 3.59 | 2.65
AVA1B2
ns
median 10.6 | 9.10
SD 3.64 | 3.31
max 13.7 | 11.6
AVA1C2
****
median 2.24 | 1.07
SD 0.513 | 0.359
max 2.83 | 1.80

Figure S3

E

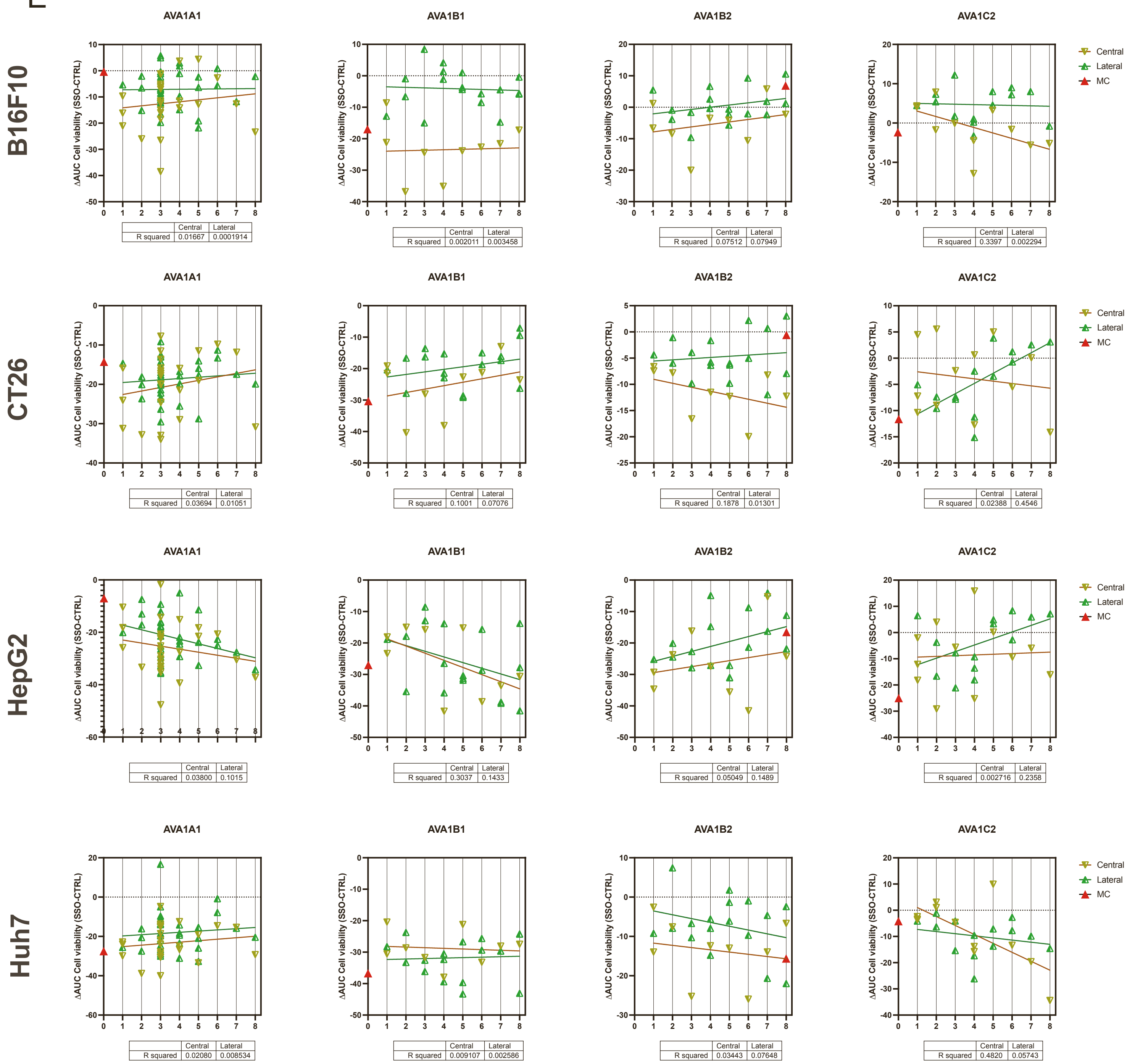

F

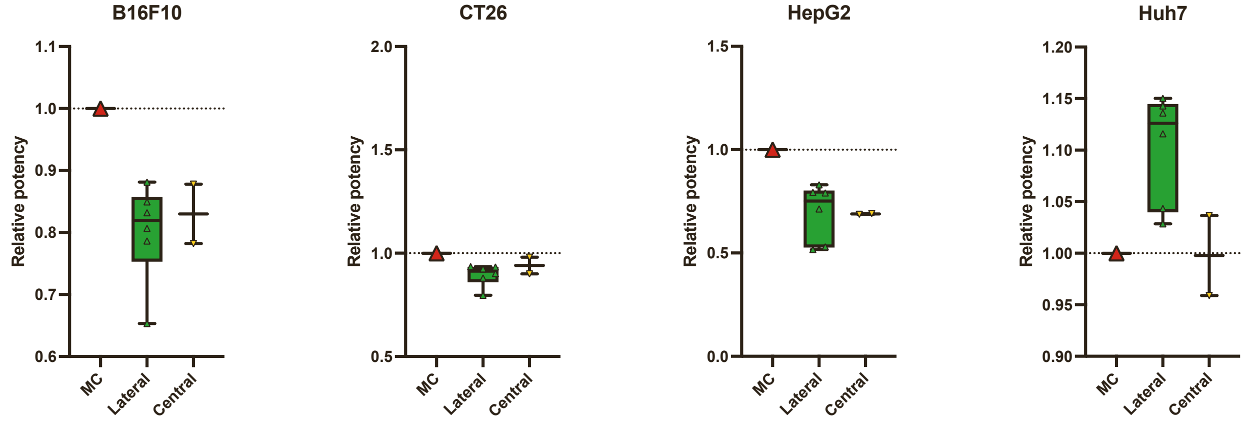

	B16F10 Lateral	B16F10 Central	CT26 Lateral	CT26 Central	HepG2 Lateral	HepG2 Central	Huh7 Lateral	Huh7 Central
median	0.819	0.830	0.913	0.940	0.752	0.690	1.13	0.998
SD	*0.0799*	*0.0678*	*0.0528*	*0.0573*	*0.139*	*0.00349*	*0.0533*	*0.0548*
max	0.881	0.878	0.935	0.981	0.830	0.692	1.15	1.04

Figure S3

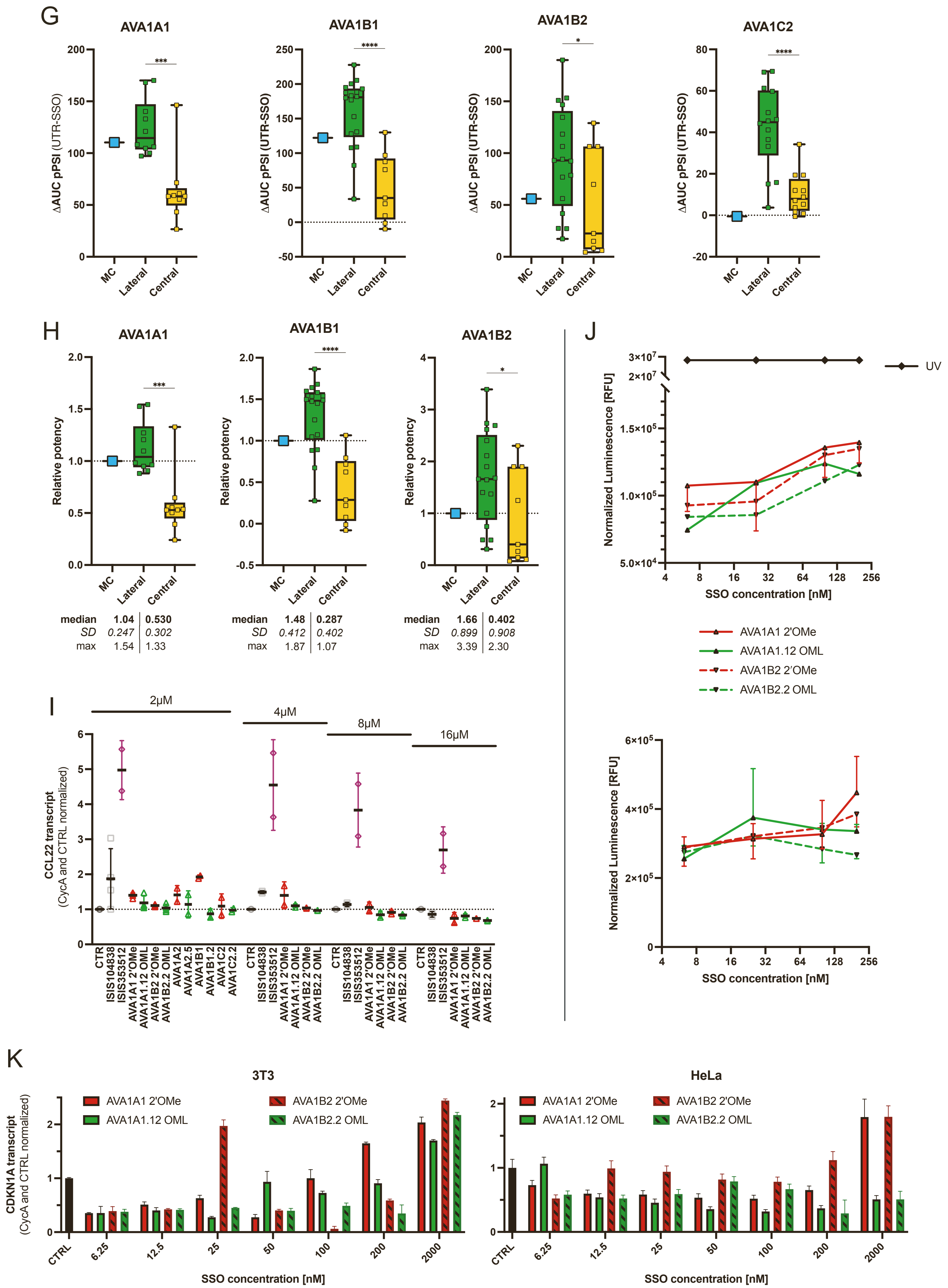
G
AVA1A1
AVA1B1
AVA1B2
AVA1C2
ΔAUC pPSI (UTR-SSO)
MC
Lateral
Central
***
****
*
****
H
AVA1A1
AVA1B1
AVA1B2
Relative potency
median 1.04 | 0.530
SD 0.247 | 0.302
max 1.54 | 1.33
median 1.48 | 0.287
SD 0.412 | 0.402
max 1.87 | 1.07
median 1.66 | 0.402
SD 0.899 | 0.908
max 3.39 | 2.30
I
2µM
4µM
8µM
16µM
CCL22 transcript
(CycA and CTRL normalized)
CTR
ISIS104838
ISIS353512
AVA1A1 2'OMe
AVA1A1.12 OML
AVA1B2 2'OMe
AVA1B2.2 OML
AVA1A2
AVA1A2.5
AVA1B1
AVA1B1.2
AVA1C2
AVA1C2.2
J
UV
Normalized Luminescence [RFU]
SSO concentration [nM]
AVA1A1 2'OMe
AVA1A1.12 OML
AVA1B2 2'OMe
AVA1B2.2 OML
K
3T3
HeLa
CDKN1A transcript
(CycA and CTRL normalized)
CTRL
SSO concentration [nM]

FIGURE S3

A. Schematics of the AVA1s target sites and their mixmers modifications by lateral and central MPCs. LNA substituted ribose are depicted in purple. For every number of substituted LNAs, there is at least one MIXMER with a Lateral MPC and a Central MPC. A maximum 6 substituted LNAs at 5' was allowed for a lateral MPC, with the remaining LNAs (if any) placed at 3', to avoid confounding as a central MPC. Scrambled sequences are the reverse-complement of the AVA1 counterparts (e.g. AVA1B1 has a non- targeting reverse-complement called NC/AVA1B1 or cAVA1B1).
B. Model summary for mixed-effects model mean ΔAUC pPSI ~ pF1*pF2*pF3 + pMid + pL3*pL2*pL1 + (oligo type| cell type), where the explanatory variables were the binary variables per SSO based on the presence or absence of LNA on each of the first and last three positions of the ASO (pF1, pF2, pF3, pL3, pL2, and pL1), an additional variable that counts the number of LNAs between the first and last three positions (pMid) and their interactions. The weights derived from this model was used to define a quantitative rule to differentiate SSOs with "lateral" and "central" MPC.
C. ΔAUC plots for AVA1A1, AVA1B1, AVA1B2 and AVA1C2 mixmers (OML), clustered by (refined) Lateral and Central MPCs, and their respective monochemistries (2'OMe) from the secondary screen in B16F10, Huh7, HepG2 NATURA cells. Refer to Fig. 3A legend.
D. ΔAUC of cell viability from the screening represented in figure 3A, clustered by LNA residues in each oligo. Each dot represents a single SSO in biological duplicate. Linear regression R2 is reported for Central and Lateral MPC. MC stands for MonoChemistry.
E. Relative exon-skipping potency of OML compared to their 2'OMe counterpart (MonoChemistry, fold change). The data was obtained in the experiment described in panel 3A. Each dot represents the average of biological duplicates for a single oligo. Median, SD and maximum are shown for lateral and central MPC clusters. Unpaired, nonparametric Mann-Whitney test (one-tailed) was performed between Lateral and Central MPC.
F. Relative exon-skipping potency of AVA1A2 OML combinations compared to AVA1A2 2'OMe. Refer to panel S3D.
G. ΔAUC plot for AVA1A1, AVA1B1, AVA1B2 and AVA1C2 mixmers (MOL), clustered by (refined) Lateral and Central MPCs, and their respective 2'MOE oligos from the secondary screen in Huh7 NATURA cells in CEM conditions (free uptake). Refer to Fig. 3C legend.
H. Relative potency (fold change) of MOL mixmer compared to 2'MOE. Data obtained from panel S3F. AVA1C2 relative potency cannot be calculated as the AVA1C2 2'MOE monochemistry did not show any exon skipping.
I. CCL22 RT-qPCR values of the best performing mixmer SSOs compared to their monochemistry counterparts. The SSO are incubated with BJAB cells for 48 hours. ISIS104838 and ISIS353512 are used as negative and positive control respectively to elicit CCL22 transcriptional activation. Each dot represents a distinct biological replicate.
J. Caspase-3/7 activity assay in HeLa (above) and 3T3 (below) transfected with monochemistry and lateral MPC SSOs. The assay was performed 16 hours post-transfection, and it is the result of two replicates. UV irradiation was used as positive control for Caspase activation.
K. RT-qPCR CDKN1A expression in 3T3 cells (normalized to Cyclophillin A housekeeper and untransfected cells) collected at 16 hours post-transfection with SSOs in HeLa and 3T3.

Figure S4

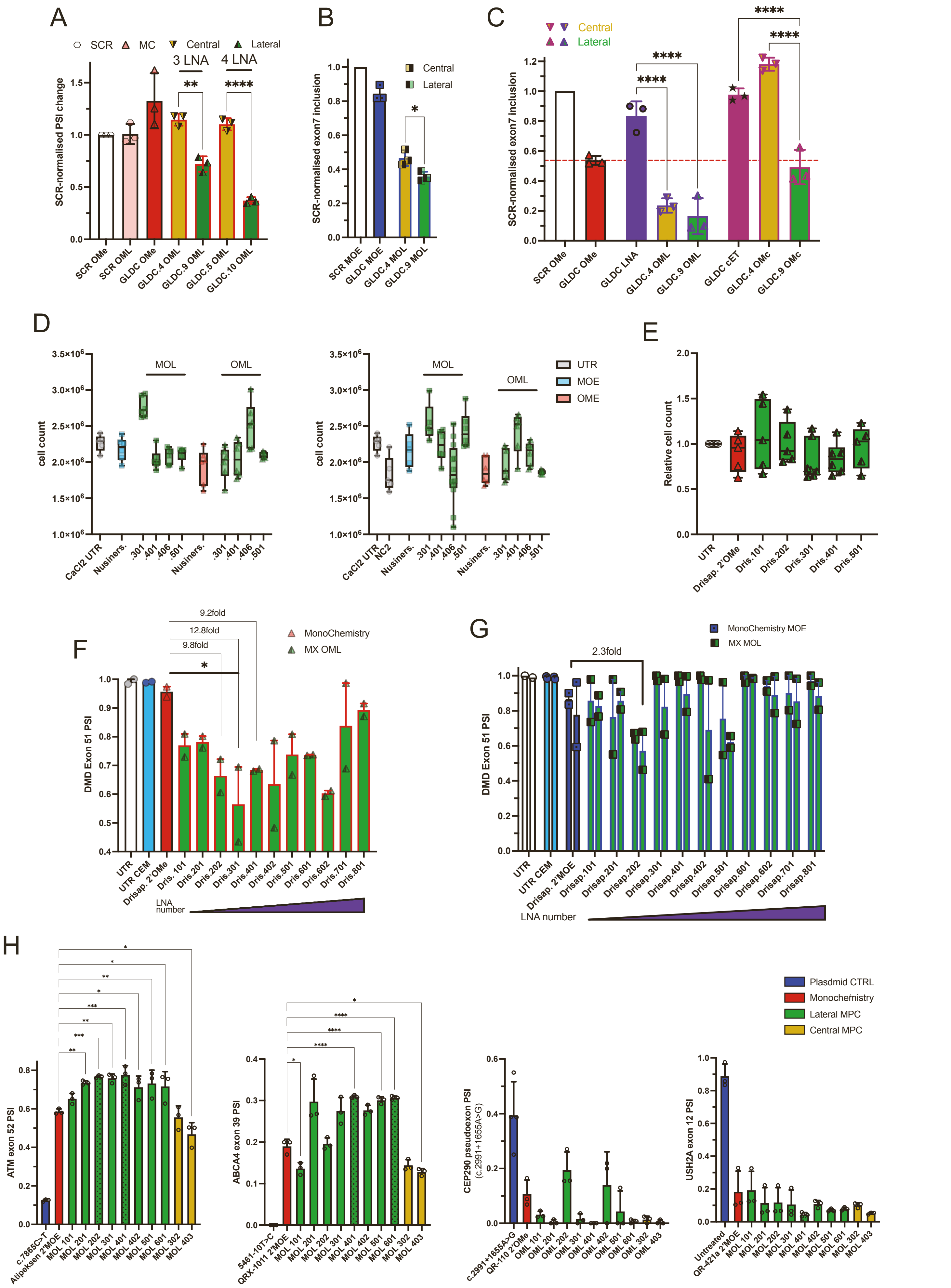
A
SCR
MC
Central
Lateral
3 LNA
4 LNA
SCR-normalised PSI change
SCR OMe
SCR OML
GLDC OMe
GLDC.4 OML
GLDC.9 OML
GLDC.5 OML
GLDC.10 OML
B
Central
Lateral
SCR-normalised exon7 inclusion
SCR MOE
GLDC MOE
GLDC.4 MOL
GLDC.9 MOL
C
Central
Lateral
SCR-normalised exon7 inclusion
SCR OMe
GLDC OMe
GLDC LNA
GLDC.4 OML
GLDC.9 OML
GLDC cET
GLDC.4 OMc
GLDC.9 OMc
D
MOL
OML
cell count
CaCl2 UTR
Nusiners.
.301
.401
.406
.501
NC2
UTR
MOE
OME
E
Relative cell count
UTR
Drisap. 2'OMe
Dris.101
Dris.202
Dris.301
Dris.401
Dris.501
F
9.2fold
12.8fold
9.8fold
MonoChemistry
MX OML
DMD Exon 51 PSI
UTR
UTR CEM
Drisap. 2'OMe
Dris. 101
Dris.201
Dris.202
Dris.301
Dris.401
Dris.402
Dris.501
Dris.601
Dris.602
Dris.701
Dris.801
LNA number
G
MonoChemistry MOE
MX MOL
2.3fold
DMD Exon 51 PSI
UTR
UTR CEM
Drisap. 2'MOE
Drisap.101
Drisap.201
Drisap.202
Drisap.301
Drisap.401
Drisap.402
Drisap.501
Drisap.601
Drisap.602
Drisap.701
Drisap.801
LNA number
H
Plasdmid CTRL
Monochemistry
Lateral MPC
Central MPC
ATM exon 52 PSI
c.7865C>T
Atipeksen 2'MOE
MOL 101
MOL 201
MOL 202
MOL 301
MOL 401
MOL 402
MOL 501
MOL 601
MOL 302
MOL 403
ABCA4 exon 39 PSI
5461-10T>C
QRX-1011 2'MOE
CEP290 pseudoexon PSI (c.2991+1655A>G)
c.2991+1655A>G
QR-110 2'OMe
OML 101
OML 201
OML 202
OML 301
OML 401
OML 402
OML 501
OML 601
OML 302
OML 403
USH2A exon 12 PSI
Untreated
QR-421a 2'MOE

FIGS4

A. PSI change of the exon targeted by the GLDC SSO in 2'OMe monochemistry or OML mixmer upon transfection with 5 nM of SSO in A549 cells. Each dot represents a biological replicate.
B. PSI change of the exon targeted by the GLDC SSO in 2'MOE monochemistry or MOL mixmer upon transfection at 5nM in A459 cells. Each dot represents a biological replicate.
C. PSI change of the exon targeted by the GLDC SSO in 2'OMe, LNA, or cET monochemistry compared to OML and OMc (OMe+cET) mixmer upon transfection at 25nM in A459 cells. Each dot represents a biological replicate.
D. Live cell count of SMN1 KO motoneurons after incubation with 100nM (left panel) and 250nM (right panel) of SSOs for 48 hours.
E. Relative live cell count of RH30 cells 24 hours post-transfection with 50nM of SSO.
F. PSI of DMD exon 51 upon incubation with Drisapersen (4μM, 2'OMe) and its mixmer permutations in RH30 cells for 48 hours in CEM. Fold-change of mixmer chemistries compared to monochemistry skipping is shown above the best performing SSOs. Each dot represents a biological replicate with a technical duplicate for each replicate. One-way ANOVA statistical analysis was carried out.
G. PSI of DMD exon 51 upon incubation with Drisapersen (4μM, 2'MOE) and its mixmer permutations in RH30 cells for 48 hours in CEM. Fold-change of mixmer chemistries compared to monochemistry skipping is shown above the best performing SSOs. Each dot represents a biological duplicate with a technical duplicate each replicate.
H. PSI of the exons in the minigenes described in figure 4D, 4E, 4F, 4G transfected with their respective targeting SSOs at 20nM (293T cells, 24h post-transfection). Only Monochemistry vs mixed chemistry significance is shown (One-way ANOVA).
